# Hybrid sampling chemometric strategy for accurate and sustainable quantification of co-administered β-lactam antibiotics

**DOI:** 10.1186/s13065-025-01695-9

**Published:** 2025-12-24

**Authors:** Shymaa S. Soliman, Mona A. Abdel Rahman

**Affiliations:** Analytical Chemistry Department, Faculty of Pharmacy, October 6 University, October 6 City, Giza 12858 Egypt

**Keywords:** Sampling techniques, Chemometrics, β-lactam analysis, Monte Carlo, Latin hypercube, Sobol sequences

## Abstract

**Background:**

Reliable chemometric models require representative and well-distributed calibration and validation datasets.

**Objective:**

The current study introduces a novel framework integrating diverse sampling strategies with multivariate modeling for the simultaneous UV–Vis quantification of aztreonam (AZM) and meropenem (MPM).

**Methods:**

Three sampling techniques, including Monte Carlo (MC), Latin Hypercube Sampling (LHS), and Sobol Sequences (SS), were systematically evaluated in combination with Partial Least Squares (PLS), Genetic Algorithm–Assisted PLS (GA-PLS), and Artificial Neural Networks (ANN). Response surface strategy was followed for each sampling technique to assess their space coverage and points’ distribution across the experimental domain. Moreover, a hybrid variable-selection approach, namely, Genetic Algorithm Information Complexity–Partial Least Squares (GA-ICOMP-PLS), was also introduced to optimize PLS model variables. Different validation techniques, including nested cross-validation, Y-randomization tests, and noise-profiling, have been followed to ensure the models’ reliability.

**Results:**

The ANN model trained with the SS technique achieved the highest predictive accuracy, reducing RMSE by 2.6% for AZM and 39.9% for MPM compared to standard PLS. However, LHS-GA-PLS delivered low prediction errors (22.8% AZM and 5.7% MPM), while MC-PLS showed lower consistency due to non-uniform sampling. GA-ICOMP-PLS further improved prediction performance for both analytes, with error reduction ranging from 35.1% to 63.6% compared to conventional PLS. Rigorous validation testing confirmed unbiased predictions and model resilience under realistic analytical conditions.

**Significance:**

This is the first study to integrate structured sampling with chemometric modeling for β-lactam analysis. The approach improved model robustness, generalization, and sustainability, achieving high green metrics of SDS (Safety Data Sheets), AGREE (Analytical Greenness metric), and BAGI (Balanced Analytical Greenness Index) values of 0.84 and 85.0, respectively. The developed method represents a reliable, efficient, and eco-friendly strategy for pharmaceutical quality control and environmental monitoring.

**Graphical Abstract:**

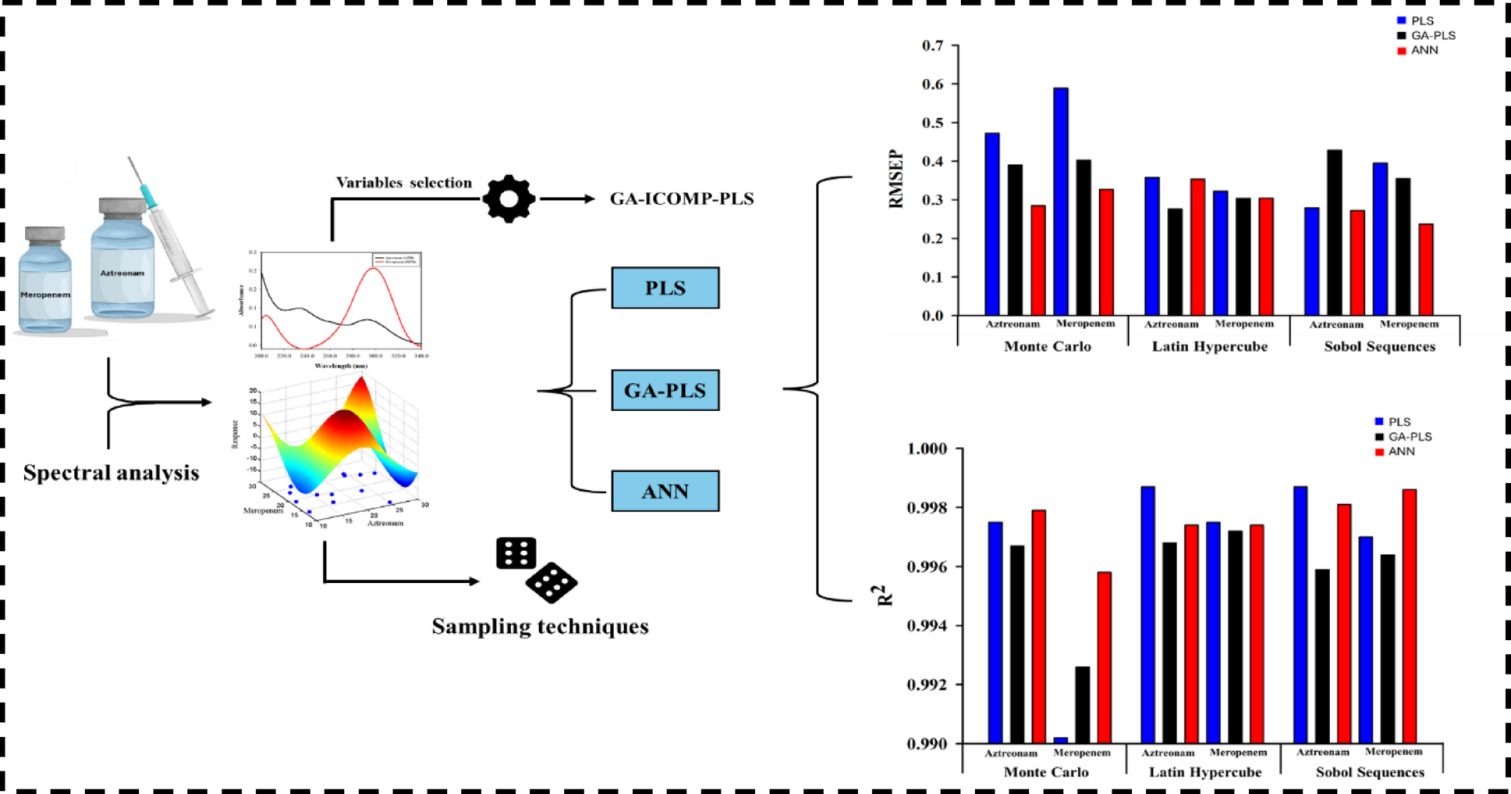

**Supplementary Information:**

The online version contains supplementary material available at 10.1186/s13065-025-01695-9.

## Introduction

Sampling is a fundamental statistical technique employed to efficiently analyze large populations or datasets, producing smaller and well-representative subsets. This approach rationalizes data analysis while maintaining resources and supporting effective decision-making without any exhaustive data collection [1, 2]. Traditional random sampling, though widely used, may hold limitations of being impractical and costly, as samples may not perfectly represent the entire population, leading to inaccurate results [3, 4]. In contrast, systematic sampling offers an equal probability of choosing each element in the population, reducing bias and improving consistency.

Advanced computational sampling techniques, including Monte Carlo (MC), Latin Hypercube Sampling (LHS), and Sobol Sequences (SS), have gained attention for their efficiency in exploring complex, multi-dimensional spaces. MC sampling relies on repetitive random distribution of data points in complex systems [5, 6]. It is known for its simplicity, flexibility, and applicability in uncertain analysis; however, it may cause clustering and gaps in the design space, especially in multi-dimensional issues. While LHS ensures uniform stratification of each variable, it provides comprehensive data coverage and enhances the method’s predictive powers [7]. However, the SS technique offers a more uniform and deterministic distribution of samples, minimizing clustering and improving the methods’ predictions [8]. By carefully selecting sampling strategies, one can generate datasets that better capture the variability in analytical systems, which is crucial for robust modeling.

Integrating these sampling strategies with regression-based chemometric techniques such as Partial Least Squares (PLS), Genetic Algorithm-Partial Least Squares (GA-PLS), and Artificial Neural Networks (ANN) has become the keystone of modern analytical chemistry. These tools allow predictive modeling even in the presence of spectral interferences, matrix effects, and experimental variability [9–13]. These computational approaches reduce the reliance on exhaustive physical experiments, aligning with green chemistry principles by lowering solvent and reagent use [12].

Sustainability is increasingly prioritized in analytical method development. Traditional protocols often use hazardous solvents, raising health and environmental concerns [14–16]. Tools such as AGREE (Analytical Greenness metric) and BAGI (Balanced Analytical Greenness Index) have emerged as reliable frameworks to quantify and visualize environmental impacts [17, 18]. They enable researchers to quantify and visualize the environmental impact of solvents and processes, fostering eco-friendly choices [19–21].

**Fig. 1:**
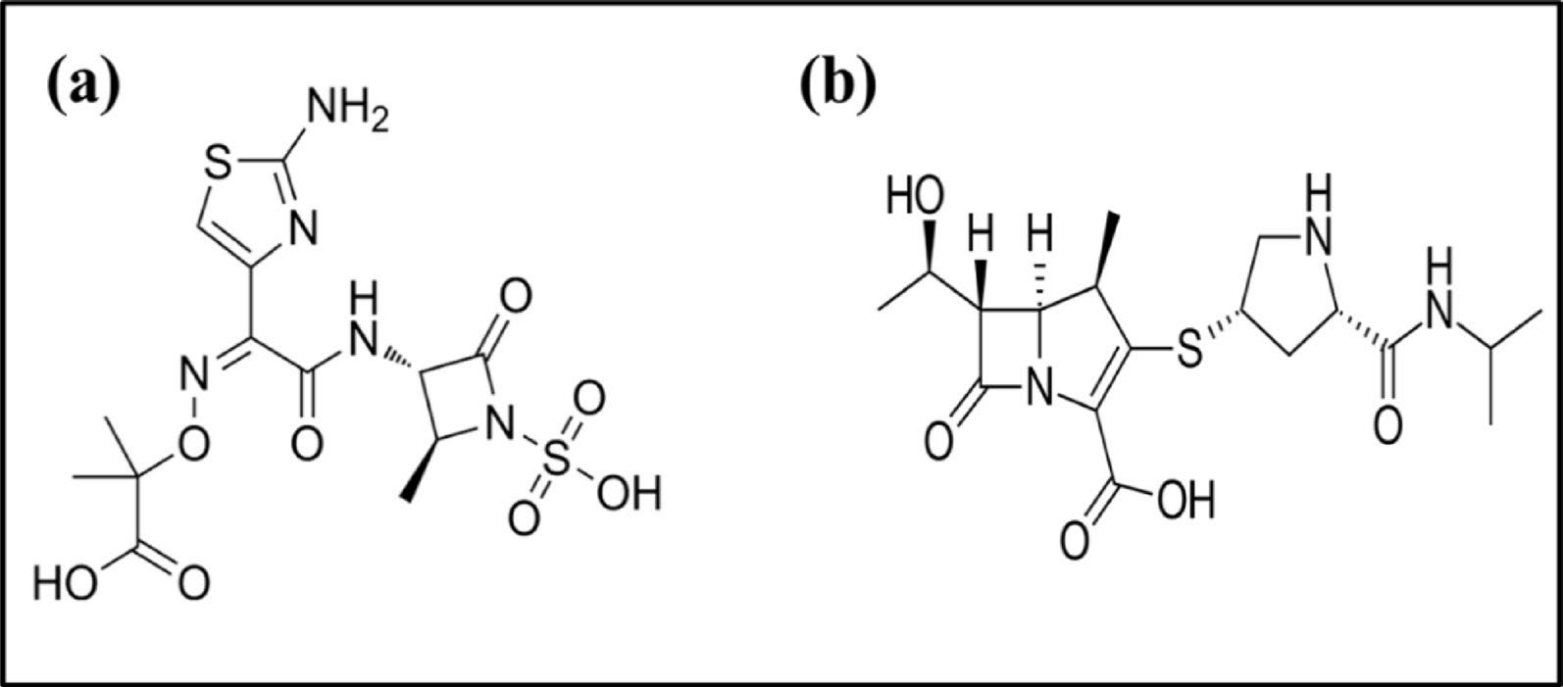
Structure of (**a**) Aztreonam and (**b**) Meropenem

Aztreonam (AZM) (Fig. 1a) chemically known as (2 S,3 S)−3-[(2Z)−2-(2-azaniumyl-1,3-thiazol-4-yl)−2-[(1-carboxy-1-methylethoxy)imino]acetamido]−2-methyl-4-oxoazetidine-1-sulfonate and meropenem (MPM) (Fig. 1b) known as (4R,5 S,6 S)−3-[(3 S,5 S)−5-(dimethylcarbamoyl)pyrrolidin-3-yl]sulfanyl-6-[(1R)−1-hydroxyethyl]−4-methyl-7-oxo-1-azabicyclo[3.2.0]hept-2-ene-2-carboxylic acid are potent co-administered β-lactam antibiotics usually used in the treatment of severe bacterial infections. Their co-administration has become increasingly significant in treating multidrug-resistant bacterial infections. While numerous studies have analyzed AZM or MPM individually [22–28] or in combination with other drugs [29–34], no study has yet applied a combination of advanced chemometric modeling with multiple sampling designs for their simultaneous quantification.

The objective of the current study is (i) to compare MC, LHS, and SS sampling strategies integrated with chemometric modeling to improve the predictive accuracy, robustness, and generalizability of multivariate models for AZM and MPM analysis, and (ii) to assess the sustainability of the developed method and solvents used using AGREE and BAGI metrics. Additionally, a comparative sustainability assessment was performed for commonly used solvents, including water, ethanol, methanol, acetonitrile, formic acid, phosphoric acid, and hydrochloric acid. These solvents were evaluated based on multiple hazard and environmental criteria derived from Safety Data Sheets (SDS). By generating diverse and representative mixture designs for calibration and validation, this study demonstrates the first chemometric approach for simultaneous AZM and MPM analysis, highlighting both methodological novelty and environmental consciousness.

## Experimental

### Instrumentation

The measurements were performed using a Shimadzu dual-beam UV–visible spectrophotometer (model 1800). A UV Probe software (version 2.32) was employed to acquire the absorption spectra. A slit width of 1.0 nm, fast scan mode, and 0.1 nm sampling interval were employed during the measurements. The chemometric modeling was performed using MATLAB^®^ version 8.1.0.604 (R2013a), equipped with the 2.1 PLS toolbox.

### Samples

#### Standard and co-packaged samples

Pure samples of AZM (98.0%) and MPM (98.0%) were obtained from Sigma Pharmaceutical Industries (Cairo, Egypt). Bi-distilled water was used as a solvent during the current work.

Aztreonam^®^ and Mirage^®^ medications (Batch No: 63323401 and 2308353), labeled to contain one gram of each component, were obtained from the local market.

#### Standard stock and working solutions

Stock solutions of AZM and MPM were individually prepared at a concentration of 1.0 mg mL^− 1^ using water as solvent. Working solutions of 100.0 µg mL^− 1^ for each drug were then obtained by the appropriate dilution of the corresponding stock solutions.

### Procedures

#### Spectral characteristics and models’ construction

A multilevel multifactorial design of different levels coded as −2, −1, 0, 1, and 2 was followed for calibration modeling. The latter held twenty-five mixtures of both drugs in a concentration range of 10.0–30.0 µg mL^− 1^. Then the absorption spectra of these mixtures were recorded against water as a blank over the 200.0–400.0 nm range, then exported to the MATLAB^®^ (R2013a) program, mean-centered, and analyzed using the chemometric models: PLS, GA-PLS, and ANN. The 200.0–335.0 nm range was used for the acquisition of data. The optimum number of latent variables (LVs), configuration parameters, and Plackett–Burman design were chosen for models’ optimization.

#### Sampling techniques

Three computational sampling techniques (MC, LHS, and SS) were employed to generate diverse and representative datasets for chemometric modeling.

##### Monte Carlo technique (MC)

Monte Carlo is a stochastic sampling process that randomly distributes sample points across the design space to approximate the behavior of complex systems. It assumes independent sampling from a uniform or specified distribution [35]. Each sample point can be represented as: *x*_*i*_ = *x*_min_ +* r*_*i*_ (*x*_max_ – *x*_min_) where *x*_*i*_ is the sampled value generated by the MC process, *r*_*i*_ is a uniform random number, *x*_*min*_ and *x*_*max*_ are the minimum and maximum limits of the variable being sampled, respectively. Using MC helps to generate random values within a specified range, ensuring that each variable’s sampling stays within its defined experimental limits. Detailed procedure and implementation steps of the MC sampling method are further described in the Supplementary Material File.

##### Latin hypercube sampling (LHS)

Latin hypercube sampling is an arithmetical technique that divides each variable’s range into equal intervals and randomly selects one value from each interval, ensuring uniform coverage of the entire parameter space [36]. This stratified approach reduces clustering and improves representativeness. Each sample is generated as: $\:{x}_{i}=\:\:{x}_{min}\:+\:\frac{{k}_{i}-\:{r}_{i}}{N}\:$(*x*_max_ –* x*_min_), where* k*_*i*_ is the stratum index, *r*_*i*_ is a uniform random number, N is the total number of sampling intervals or divisions within the range of the variable,* x*_*min*_ and* x*_*max*_ represent the lower and upper bounds of the variable range, respectively. Further details of the LHS procedure are included in the Supplementary Material File.

##### Sobol sequence (SS)

Sobol sequence is a quasi-random sequence that provides uniform coverage of multidimensional spaces, minimizing gaps or clustering [37]. Each point is deterministically generated as $\:{x}_{i}^{\left(j\right)}=\:{b}_{1}$υ_*1, j*_^⊕^_,_ $\:{b}_{2}$υ_*2*_, _*j*_^⊕^_, ……,_ using bitwise operations (⊕) on precomputed direction numbers (detailed in the Supplementary Material File).

Overall, this entire workflow was designed to ensure robust, reproducible, and sustainable chemometric modeling for simultaneous quantification of AZM and MPM.

#### Models’ validation

##### Spectral data collection

Absorbance data for AZM and MPM were collected in the range of 200.0–335.0 nm using a UV-visible spectrophotometer. Calibration and validation mixtures were prepared with concentrations ranging from 10.0 to 30.0 µg mL^− 1^ for both drugs.

##### Data pre-treatment

Spectral data for AZM and MPM were preprocessed using mean centering, a linear transformation that subtracts the mean of each variable to remove offsets and improve numerical stability.

##### Experimental design

The calibration and validation mixtures were designed according to a Plackett–Burman screening design, which systematically varied the concentrations of AZM and MPM to ensure representative coverage of the experimental domain.

##### Modeling of the validation set

Thirteen mixtures of random concentrations were designed using the MC, LHS, and SS sampling techniques and used as a validation set. These techniques were implemented using built-in scripts found in MATLAB^®^ R2013a, then the obtained results were later employed for identifying the best sampling strategy that offers the optimum responses in terms of predictive accuracy, reduced bias, and improved generalizability.

##### Construction and optimization of chemometric models

Various multivariate models, PLS, GA-PLS, and ANN, were developed and optimized for analyzing AZM and MPM in bulk and dosage forms. Regarding the PLS model, the data interpretability and model optimization were carried out using the leave-one-out cross-validation method, where the ideal number of latent variables (LVs) was selected for model accuracy and simplicity. The GA-PLS algorithm was employed to enhance the PLS predictive capability and identify the most informative variables. Different simulated steps were performed for optimum variable selection, where different parameters for the GA model, including population size, subset size, and mutation rate, were adjusted to select the best-performing wavelengths. Additionally, the ANN model was developed for nonlinear modeling and pattern recognition. The backpropagation algorithm and various iterative weights were used for ANN training and error reduction. Model flexibility and efficiency were maintained using the Purelin-to-Purelin transfer function.

##### Evaluation of training and testing datasets

Different parameters, such as the recovery percent (%R), standard deviation (SD), and relative standard deviation (% RSD), were estimated to assess the models’ accuracy. The models’ accuracy was gauged using the root mean square error of calibration (RMSEC) and the root mean square error of cross-validation (RMSECV), where the RMSEC assesses the models’ fitness with the training set, while the RMSECV appraises the models’ generalization regarding hidden data. However, the root mean square error of prediction (RMSEP) was used for assessing the models’ practical efficacy using an independent validation set. The reproducibility and robustness of the developed models were checked by reanalyzing the validation set in an independent laboratory. This approach mimics real-world scenarios in which predictive models are implemented across different laboratories or quality control settings using various spectroscopic techniques, thus allowing for bias reduction and models’ reliability.

##### Models generalization

To rigorously assess the generalization ability and prevent information leakage, nested cross-validation was subsequently performed, with inner folds used for hyperparameter optimization and outer folds for unbiased performance evaluation. External validation was further conducted on an independent set of 13 mixtures to evaluate the models’ real-world predictive ability. Model performance was quantified using RMSECV and RMSEP, with 95% confidence intervals obtained via bootstrap resampling to account for variability and uncertainty. To ensure that the observed predictive ability was not due to chance correlations, Y-randomization tests were performed. The applicability domain (AD) was assessed using Williams plots, which display standardized residuals against leverage values, with the critical leverage limit (h^*^) defining the reliable prediction space and confirming that all predictions lie within the model’s interpolation domain.

#### Chemometric optimization using novel variable selection strategy (GA_ICOMP_PLS)

In the current study, a new variable selection technique named Genetic Algorithm Information Complexity for Partial Least Squares (GA-ICOMP-PLS) was utilized, where a binary-coded GA with an information-theoretical fitness function was integrated to optimize the variables implemented into the PLS regression model. Unlike the conventional GA-PLS method, the GA-ICOMP-PLS employs the Information Complexity criterion (ICOMP) to balance model accuracy and complexity as follows: ICOMP = − 2 log L + 2 C(Σ), where L and C(Σ) represent the likelihood function and the complexity penalty, respectively. The term − 2 log L quantifies model fit, and was computed under the assumption of normally distributed residuals, as follows: $\:-2\:log\:L=n\mathrm{ln}\left(2\pi\:{\sigma\:}^{^2}\right)+\:\frac{{\parallel\:\mathrm{y}-\mathrm{y}^\parallel\:}^{2}}{{{\upsigma\:}}^{^2}}\:$, where n is the estimated residual variance, y is the reference response vector, and y ^ represents the predicted responses from the PLS model. The covariance complexity term is derived from the covariance matrix of the PLS regression residuals, and computed according to Bozdogan’s information-complexity formulation: $\:\mathrm{C}\left({\Sigma\:}\right)=\:\frac{1}{2}\:\left[tr({\Sigma\:}{)}^{2}-\:tr({{\Sigma\:}}^{2}\:)\right]$. This term penalizes models with higher parameter interdependence and greater covariance structure complexity, encouraging a more parsimonious latent-variable representation in the PLS model. This ensures that selected spectral variables not only minimize prediction error but also result in stable and well-conditioned models.

The algorithm was performed over 50 generations and 30 population sizes, using uniform crossover and a 1% bit-flip mutation rate. The chromosome yielding the lowest ICOMP score was selected as the optimal solution. The performance of GA-ICOMP-PLS was benchmarked against both conventional GA-PLS and full-spectrum PLS models to evaluate its effectiveness in variable reduction and prediction robustness.

#### Analysis of dosage forms

Aztreonam^®^ and Mirage^®^ dry powders (labeled to contain 1.0 g of AZM and MPM, respectively) were purchased from the local market. An amount equivalent to 100 mg of each drug was weighed into the same volumetric flask and sonicated in distilled water for 10 min, then filtered through a 0.45 μm membrane filter. Further dilutions were made to attain concentrations within the established calibration range, and the analysis was carried out according to the models’ general procedures. The five determinations’ average was employed for assessing the precision of the constructed models.

#### Comparative studies between sampling techniques and models

To study the influence of sampling strategies on models’ robustness and predictive accuracy, three distinct sampling techniques, such as MC, LHS, and SS, were applied to generate reliable and more consistent synthetic mixtures. Each technique was utilized to create 13 validation mixtures, consistent with the experimental design previously optimized for chemometric modeling. All mixture designs were constructed to span the multidimensional concentration space as uniformly as possible. In the MC technique, the samples were randomly selected within the defined concentration limits for each analyte, while in the LHS technique, each variable’s concentration range was stratified into equal intervals to ensure a more uniform and representative sample coverage. The SS technique generated sample designs with obvious space-filling characteristics, particularly in high-dimensional data structures. These techniques were implemented in MATLAB^®^, and the developed mixtures were subjected to spectrophotometric analysis over the 200.0–400.0 nm wavelength range. The resulting absorbance spectra were then used as inputs for the chemometric modeling approaches: PLS, GA-PLS, and ANN. Different evaluation metrics, including the RMSEP, correlation coefficient (R²), %R, and %RSD, were calculated for techniques evaluation.

## Results and discussion

Integrating advanced sampling strategies with chemometric-based techniques has become the cornerstone of modern analysis, especially in developing more predictive, robust, and reliable models. This synergistic approach enhances the efficiency of experimental design, reduces resource consumption, and facilitates the generation of more representative datasets. During the current study, various sampling strategies, MC, LHS, and SS, were evaluated and compared to identify the approach that provides the best efficacy in terms of predictive accuracy, reduced bias, and improved generalizability. As a case study, these sampling methods were integrated with different chemometric modeling techniques, including PLS, GA-PLS, and ANN, to facilitate the quantitative analysis of AZM and MPM across different sample matrices. This integrated framework aimed to illustrate the effectiveness of combining advanced sampling with multivariate calibration for improved analytical outcomes (Scheme 2).

**Scheme 1:**
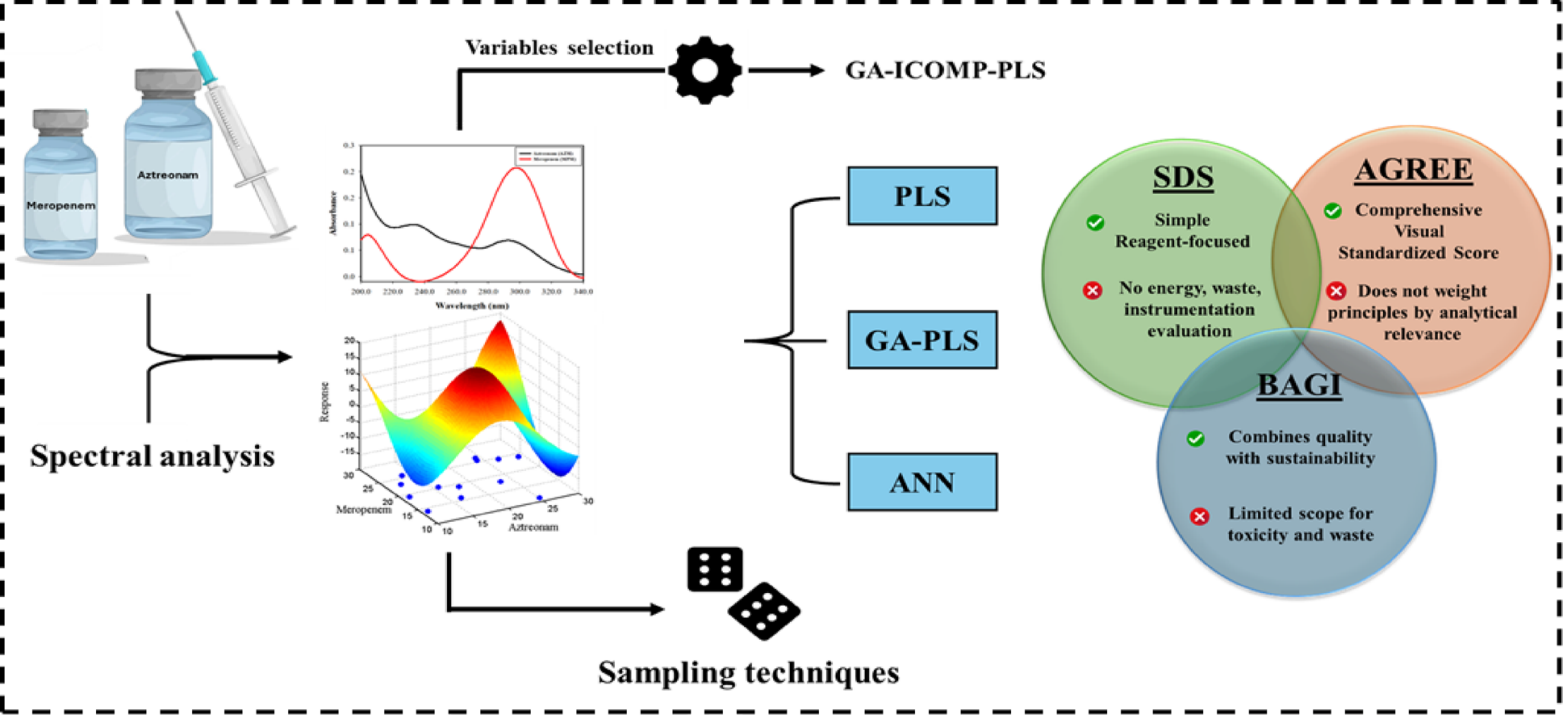
Schematic illustration of the integrated chemometric–greenness framework for the simultaneous quantification of aztreonam and meropenem. Sampling and modeling strategies (PLS, GA-PLS, ANN) were integrated with greenness evaluation tools (SDS, AGREE, BAGI). The Venn diagram highlights the comparative strengths and limitations of the three greenness assessment tools, emphasizing their complementary roles in evaluating analytical sustainability

### UV spectral interpretation

**Fig. 2:**
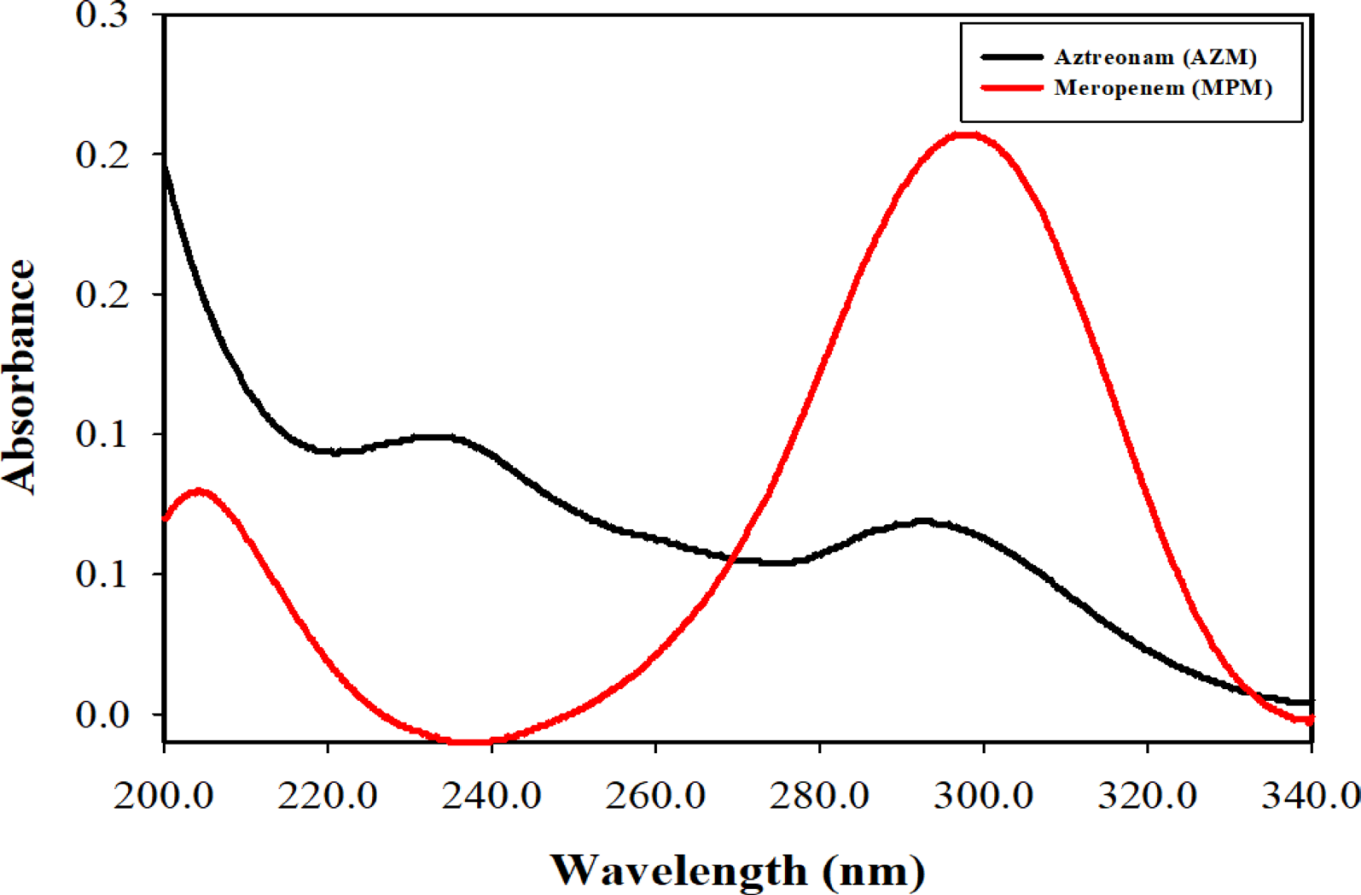
Zero-order absorption spectra of aztreonam and meropenem, 10.0 µg mL^− 1^ each, using water as a blank

The spectra of AZM and MPM were scanned over the wavelength range of 200.0–400.0 nm to identify regions with optimal absorption features. A fast scan speed was selected, where an interval of 0.1 nm was chosen for acquiring data. Upon examining the UV spectra of AZM and MPM, a noticeable overlap appeared (Fig. 2) that may be attributed to their corresponding structural chromophores. AZM exhibited strong absorption in the 200.0–250.0 nm region, attributed to π→π* electronic transitions of the β-lactam ring and aromatic thiazole moiety, with a minor extension up to 340.0 nm, arising from n→π* transitions associated with amide and sulfonate functionalities. In contrast, MPM displayed a dominant broad band in the 260.0–330.0 nm range, mainly due to n→π* transitions of its conjugated β-lactam and pyrrolidine carbonyl systems, along with minor contributions from aliphatic substituents below 230.0 nm.

The obvious overlap within the 200.0–340.0 nm region introduced strong spectral collinearity, making univariate analysis insufficient for accurate quantification. Consequently, multivariate chemometric models such as PLS, GA–PLS, and ANN were employed to extract relevant spectral variance and resolve the two analytes simultaneously, linking the mathematical treatment directly to their molecular electronic transitions. A measuring wavelength range of 200.0–335.0 nm (676 points) was used for the construction of the developed models, while wavelengths below 200.0 nm and above 335.0 nm were excluded to prevent models’ disturbances. The constructed models, especially GA–PLS and ANN, demonstrated superior performance, effectively capturing subtle hidden nonlinearities and selective wavelengths within the overlapping region, thereby improving both predictive accuracy and chemical interpretability.

### Construction and optimization of the chemometric models

**Fig. 3:**
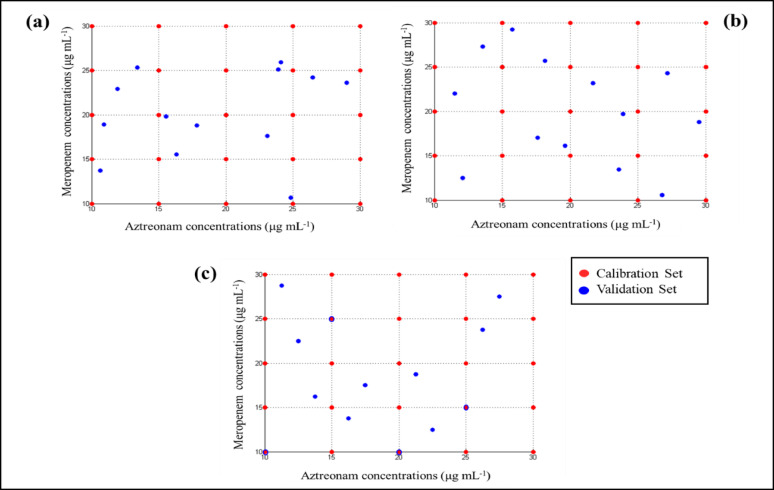
2D space-filling scattered plot indicating the validation set that was designed using **a** Monte Carlo, **b** Latin hypercube sampling, and **c** Sobol sequence

Twenty-five mixtures were prepared using a multilevel multifactorial design and used as the training set (Supp. Table S1). The concentration values ranged from 10.0 to 30.0 µg mL^− 1^ for both drugs and were analyzed using the developed models. The sampling techniques: MC, LHS, and SS, were later used for designing the validation sets (Supp. Table S1). Each set was constructed according to the corresponding strategy of the utilized sampling technique. Thirteen mixtures with different concentrations were prepared based on variable numbers and modeled mixtures in the calibration set (Fig. 3) and then analyzed using the same analytical procedures. The division of calibration and validation sets was selected in accordance with commonly reported chemometric practices, where 20 – 30 calibration mixtures are typically sufficient to obtain stable and representative multivariate models. The 2:1 calibration-to-validation ratio ensures adequate model training and unbiased external evaluation. Generating samples using MC, LHS, and SS techniques was done to provide uniform coverage of the experimental domain and to guarantee that both calibration and validation mixtures spanned the same spectral and compositional regions.

A cross-validation method was used for PLS construction, where three LVs were optimum for model construction (Supp. Figure S1). Small values of RMSEC were obtained, as shown in Table 1, indicating good model accuracy and simplicity. Moreover, the predictivity of the PLS model was further enhanced using the GA tool. Different populations were investigated to recognize the optimal spectral regions and LVs, thus excluding redundant wavelengths. The GA parameters were optimally designed (Supp. Table S2), where the data input was reduced by approximately 59.3% and used later for PLS construction. Despite the consistent remaining of LVs at three (Supp. Figure S1), the RMSEC values of the GA-PLS model were much lower than those of the PLS model (Table 1), indicating better model accuracy and reliability.

**Fig. 4:**
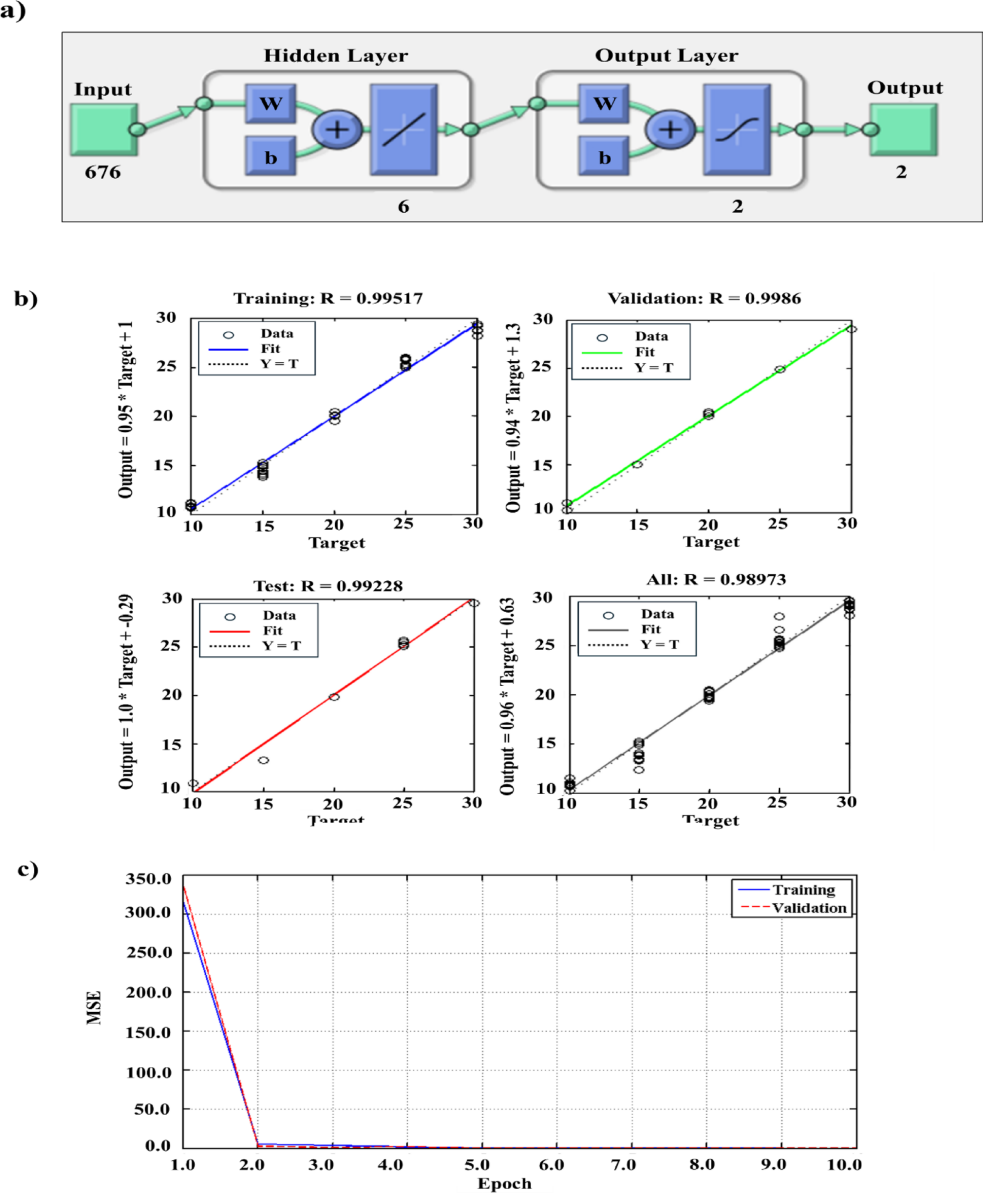
**a** The architecture of the ANN network used for the prediction of components’ concentrations using different layers, **b** prediction plots of the training and validation sets, and **c** learning curves of the ANN model trained using the Levenberg–Marquardt (TRAINLM) algorithm

The ANN model was constructed using the TRAINLM algorithm (Levenberg-Marquardt backpropagation) to effectively capture potential nonlinearities in the spectral data. The propagation process of the network can be expressed as: *y*^ = *f*(*W*_2_⋅* g* (*W*_1_*X* +* b*_1_) +* b*_2_) where *X* denotes the input spectral matrix, *W*_1_ and *W*_2_ are weight matrices, *b*_1_ and *b*_2_ are bias vectors, *g(.)* and *f(.)* are activation functions, and $\:\widehat{y\:}$ is the predicted concentration. The data were iterated repeatedly for optimum network training. The training minimized the MSE $\left[ {MSE = \frac{1}{N}\sum\nolimits_{{i = 1}}^{N} {\left( {y_{i} - y_{{\hat{i}}} } \right)^{2} } } \right]$ and updated parameters following the Levenberg–Marquardt rule [w_k+1_​ = w_k​_ − (J^T^ J + µI)^−1^ J^T^ *e*]. Six neurons and a maximum training limit of 400 epochs were used as ANN configurations to ensure sufficient learning capacity. Different layers were included in the ANN architecture for both drugs (Fig. 4a), and the prediction plots of both the training and validation sets showed an r close to 1.0 (Fig. 4b), demonstrating a good model prediction.

Although a maximum of 400 epochs was predefined, early stopping occurred at approximately the 5th epoch based on the validation error criterion, preventing overfitting. Learning curve analysis showed rapid error reduction and close alignment between training and validation MSE curves (Fig. 4c), indicating fast convergence and excellent generalization. Unlike linear models such as PLS and GA–PLS, which assume linear dependence between spectral intensity and analyte concentration, the ANN can precisely handle nonlinearities arising from overlapping absorption bands, matrix effects, and instrumental noise. Through iterative weight optimization and backpropagation, the network effectively minimizes residual errors and learns complex spectral-concentration relationships. This adaptive learning behavior enhances the model’s predictive accuracy and generalization, particularly for datasets exhibiting latent nonlinear interactions among wavelengths.

**Table 1:** Efficacy metrics for each model for the calibration set

Parameter		Model		
		PLS	GA-PLS	ANN
Aztreonam	Concentration range (µg mL^− 1^)	10.00–30.00		
	Slope	1.0011	1.0000	0.9868
	Intercept	−0.0581	0.0004	0.2160
	Correlation coefficient (r) ^a^	0.9989	1.0000	0.9985
	RMSEC ^b^	0.3913	0.0317	0.3287
Meropenem	Concentration range (µg mL^− 1^**)**	10.00–30.00		
	Slope	0.9992	0.9998	0.9770
	Intercept	0.0159	0.0036	0.3864
	Correlation coefficient (r) ^a^	0.9996	0.9998	0.9986
	RMSEC ^b^	0.4068	0.0949	0.1996

^a^The data correspond to the straight line outlined between predicted concentrations of each component versus actual concentrations of the calibration set

^b^Root Mean Square Error of Calibration

### Evaluation of sampling techniques and chemometric models

**Fig. 5:**
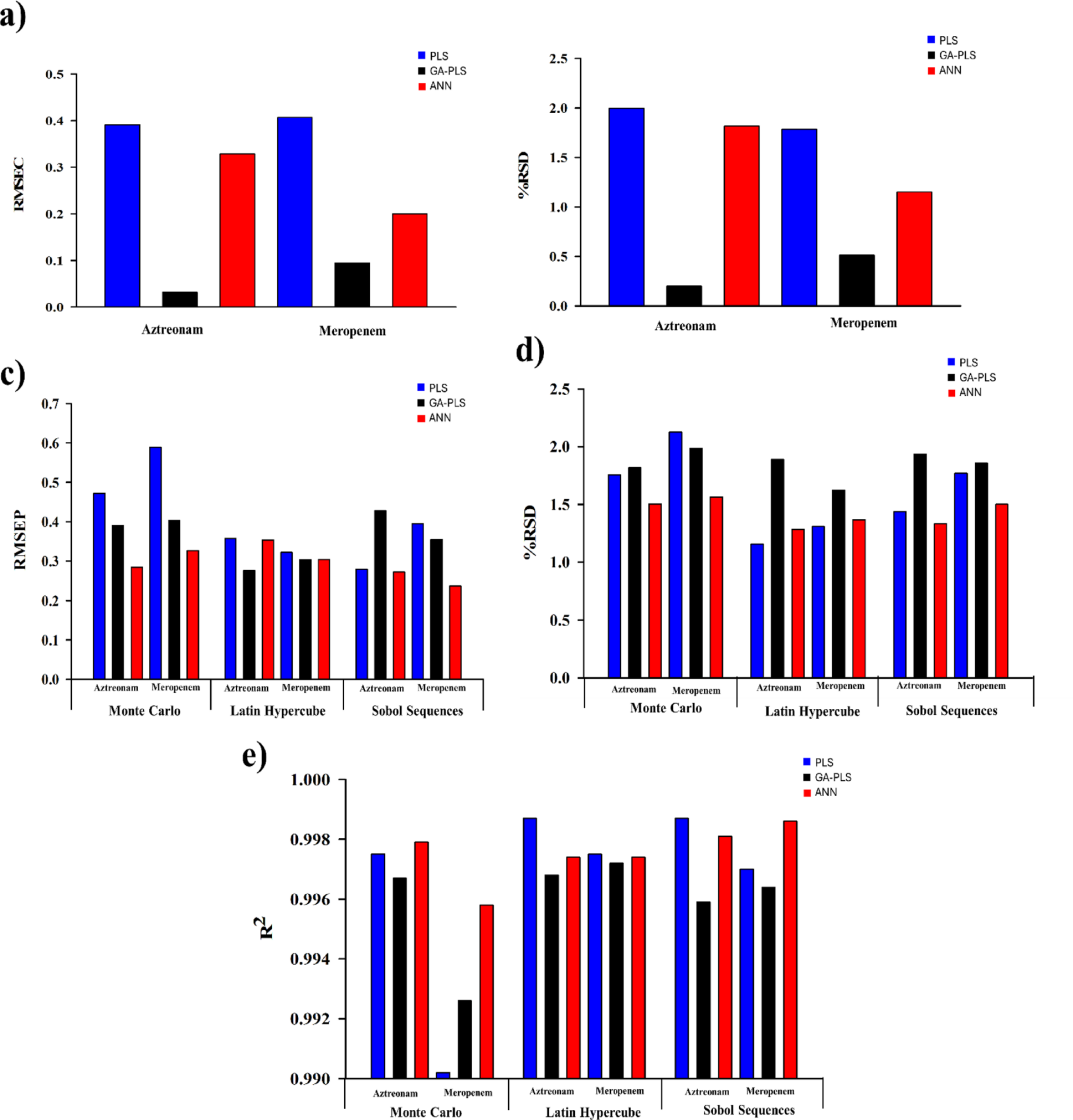
**a** The RMSEC and **b** relative standard deviation values of the studied components using the calibration models, **c** the RMSEP, **d** relative standard deviation, and **e** R^2^ values using the validation sets of different sampling techniques

Various parameters, such as the %R, %RSD, and the RMSEC, were successfully evaluated. Minimum values of RMSEP and %RSD indicated the optimal predictability and precision of the developed models (Supp. Table S3). A comparison was held between the developed models using the RMSEC and RMSEP values of the calibration and the validation sets. The study indicated the high efficiency and reliability of the models for analyzing complex mixtures. Among the tested models, GA-PLS demonstrated improved variable selection efficiency and consistently achieved the lowest RMSEC (Fig. 5a) and %RSD (Fig. 5b) values, reducing overfitting compared to standard PLS. Both models, GA-PLS and ANN, lessened the RMSE by 91.9%, 76.7%, 16.0%, and 50.9% for AZM and MPM, respectively, implying better model generalization.

### Models’ validation, robustness, and predictive performance

To verify the robustness and generalization ability of the developed chemometric models and to exclude the possibility of overfitting, multiple validation strategies were applied.

#### Nested cross-validation and external validation

All models (PLS, GA-PLS, and ANN) were evaluated using nested 5-fold cross-validation. In this framework, the inner folds were used for hyperparameter optimization (PLS latent variables, GA feature selection, ANN neuron architecture), while the outer folds assessed out-of-sample predictive performance. External validation was performed on an independent set of 13 mixtures (validation set). RMSE metrics were reported with 95% confidence intervals obtained via bootstrap resampling. The predictive performance of all models for AZM and MPM is summarized in the Supp. Table S4. GA-PLS achieved the lowest RMSECV values due to effective feature selection, while ANN models showed slightly higher errors as all spectral variables were used. RMSEP values on the external validation set confirm that all models generalize well beyond the calibration data.

#### Y-randomization test

To verify the robustness and generalization ability of the developed models and to exclude the possibility of overfitting, Y-randomization (Y-scrambling) tests were conducted by randomly shuffling the response variable while maintaining the predictor matrix constant. The resulting models exhibited drastically reduced predictive power (*r* < 0.5 and high RMSE values), confirming the absence of chance correlation. Moreover, the close agreement between RMSEC and RMSEP, along with cross-validation diagnostics, demonstrates that the models effectively captured the true analyte spectral relationships within the calibration domain.

#### Applicability domain (AD)

The reliability and prediction boundaries of the developed models were evaluated through the applicability domain (AD) concept using a Williams plot, which represents the standardized residuals against leverage values for both AZM and MPM. The critical leverage limit (h^*^) was calculated to define the model’s reliable prediction space. All calibration and validation samples were found within the accepted limits (standardized residuals less than 3 and leverage value less than h^*^), confirming the absence of influential outliers and that all samples lie within the model’s reliable prediction space (Supp. Figure S2).

#### Noise profile and sampling step

To assess data quality and ensure that model performance was not driven by oversampling or spectral noise, the signal-to-noise ratio (SNR) was evaluated across the UV range (Supp. Figure S3). The spectra demonstrated a stable SNR profile (3.6–4.4), with the highest values observed around 202.0 nm and a mild decline near 322.0 nm. Importantly, the SNR curve showed no abrupt spikes, indicating consistent instrumental performance and reliable signal capture across the full wavelength range.

To further rule out artifacts related to oversampling, the spectral data were downsampled (binned) from 0.1 nm to 0.5 and 1.0 nm steps. PLS models built on all downsampled datasets showed similar RMSEP and R² values for both AZM and MPM, confirming that predictive performance is independent of the fine spectral resolution (Supp. Table S5). Furthermore, trimming the spectra to 200.0–335.0 nm did not compromise model accuracy, indicating that the models rely on high-quality spectral signals rather than noisy regions outside the instrument’s effective bandwidth.

### Integrated comparison of sampling-model combinations

The uniformity of each sampling technique was assessed based on two metrics: the minimum pairwise distance and the grid coverage percentage. The minimum pairwise distance reflects how dispersed the points are in the design space, while the grid coverage quantifies the percentage of a discretized 2D grid that is occupied by sample points. Among the three methods, LHS revealed the highest minimum distance (0.121) and the highest grid coverage (13%), indicating superior space-filling characteristics and even distribution across the design domain. The SS also provided high coverage as LHS (13%) with slightly lower pairwise distance (0.097), suggesting more uniform but denser packing. In contrast, MC sampling produced the lowest minimum distance (0.05) and lowest grid coverage (12%), demonstrating its tendency for clustering and sparse areas. These results confirm that structured sampling methods such as LHS and SS significantly outperform random MC sampling in terms of domain representation, which is crucial for building robust and generalizable chemometric models.

**Fig. 6:**
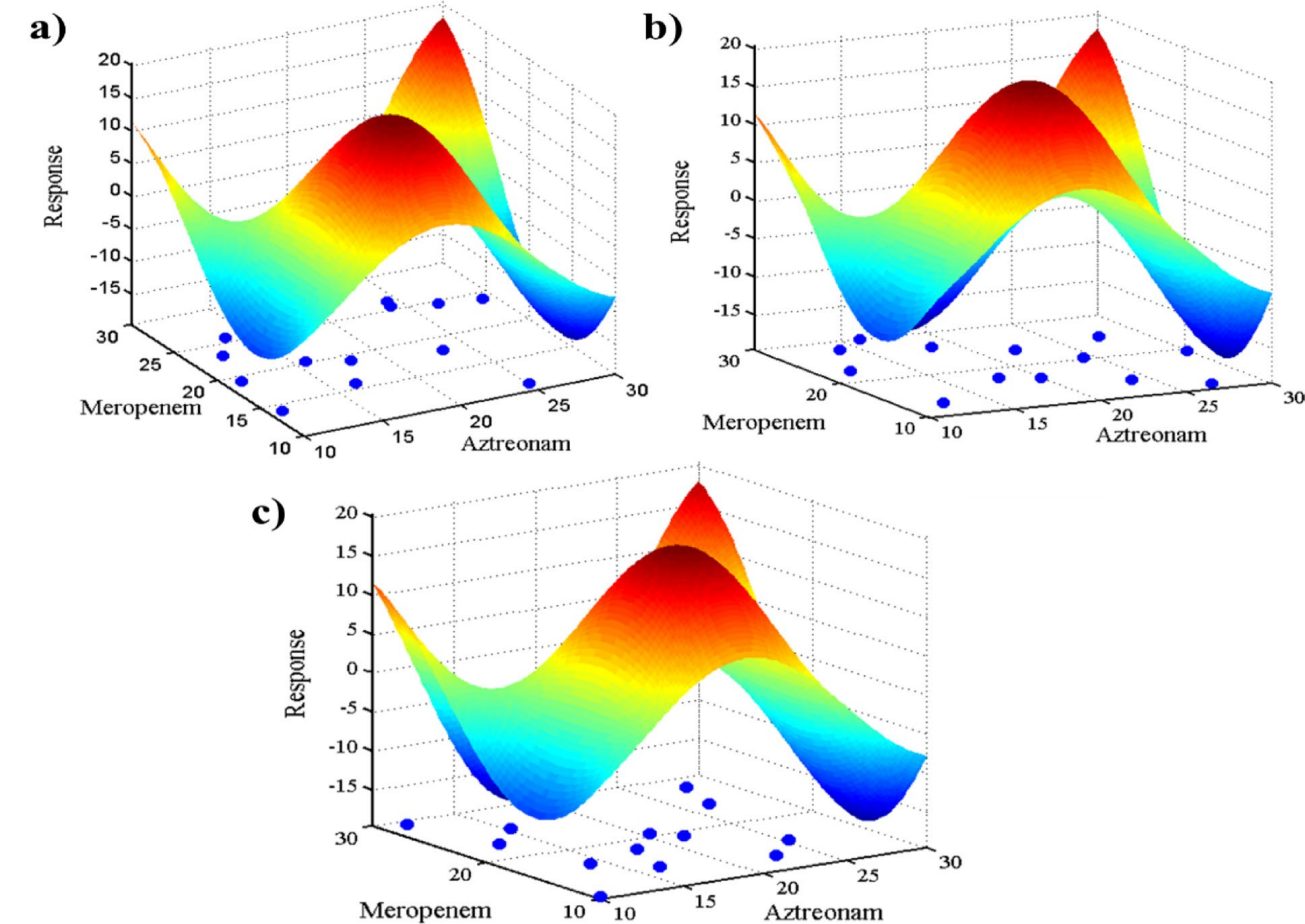
3D surface plots illustrating the distribution of sampling points generated using (**a**) Monte Carlo, (**b**) Latin Hypercube Sampling, and (**c**) Sobol Sequences over the experimental domain of aztreonam and meropenem concentrations. Blue dots represent calibration points projected onto the base of the response surface

The influence of these sampling techniques on models’ development and calibration space coverage was further evaluated using response surface visualization. As shown in Fig. 6, the MC sampling technique showed a random, non-uniform distribution of sample points with noticeable clustering and gaps (Fig. 6a). This irregular coverage negatively impacts models’ robustness and predictive power. On the contrary, the LHS technique (Fig. 6b) showed stratified and evenly distributed points through the sample space, ensuring adequate representation of each region, thus supporting models’ accuracy and reproducibility, especially for linear models such as PLS and GA-PLS. However, being a quasi-random low-discrepancy method, the SS technique exhibited a well-uniform coverage across the design space (Fig. 6c), which enhanced the generalizability of the constructed models, particularly nonlinear models such as ANN, and prevented models’ overfitting while ensuring better prediction abilities in design invisible domains.

A comprehensive comparison was made by integrating each sampling method with the three chemometric models. Various metrics (R², RMSEP, %RSD) were tabulated and visualized to identify the best combinations (Fig. 5). The ANN model, when trained with the SS sampling technique, yielded the lowest RMSEP (Fig. 5c) and %RSD (Fig. 5d) values with the highest accuracy for both AZM (R² = 0.9981) and MPM (R² = 0.9986) (Fig. 5e). This may be attributed to the uniform and quasi-random distribution of the SS-generated points, which minimizes clustering and ensures comprehensive coverage of the experimental domain. This uniformity enhances the ANN’s capability to learn complex nonlinear patterns between spectral intensities and concentration levels, improving generalization and minimizing overfitting. However, GA-PLS paired with LHS showed robust results with relatively low prediction errors (Fig. 5c). This may be due to the stratified yet randomized distribution of LHS points, which preserves data linearity and reduces collinearity among predictors. This distribution facilitates the GA’s variable selection process, leading to more representative latent structures and enhanced PLS robustness. Conversely, MC sampling combined with PLS exhibited lower efficiency. Being purely random, MC tends to yield less uniform coverage of the design space, which can introduce data clustering and reduce model stability. Collectively, these findings demonstrate that matching the sampling design to the model’s learning behavior is crucial for optimizing predictive abilities, where uniform yet structured sampling (as in LHS and SS) provides a superior foundation for both linear (GA-PLS) and nonlinear (ANN) models.

To statistically verify the observed differences among sampling techniques, the RMSEP values were evaluated using the Friedman nonparametric ANOVA test. The test indicated *p* < 0.001 for both AZM and MPM, suggesting statistically significant variations among the sampling strategies. However, the direction and magnitude of the differences were fully consistent with the observed RMSEP results, at which the ANN–SS model achieved the highest predictive accuracy and generalization ability, while GA–PLS with LHS exhibited reliable robustness and relatively low prediction errors. The PLS–MC model showed the least consistency due to the nonuniform distribution inherent to MC sampling. These findings confirm that the observed improvements in predictive abilities are statistically supported, and the overall ranking of the sampling strategies remains unchanged.

### Selecting variables using the GA-ICOMP-PLS

The GA-ICOMP-PLS algorithm was implemented in the current study to select the optimal spectral variables for the PLS regression. A complexity penalty was introduced based on the covariance structure of the prediction error. An informative subset of spectral variables was identified and used for GA construction. Unlike conventional variable selection approaches, GA-ICOMP applies the information-theoretic criterion (ICOMP) that balances model fit with structural simplicity. The resulting variable pattern reflects regions of the spectrum with the most predictive significance. This is done to avoid overfitting and variables’ redundancy, especially in collinear and high-dimensional spectral datasets. The selected wavelengths were clustered in chemically relevant regions, suggesting meaningful absorption contributions from the analytes of interest. Model activity was assessed, where prediction error values of 0.2114, 0.2522, 0.1501, 0.1742, 0.1811, and 0.1435 were yielded for AZM and MPM using MC, LHS, and SS techniques, respectively.

### Dosage form analysis and models’ statistical comparison

The developed models were utilized for determining the concentrations of AZM and MPM in their respective dosage forms. The results demonstrated high accuracy with low standard deviation values, confirming the null interference of common excipients (Table 2).

The models’ efficacy was evaluated and statistically assessed using a reported HPLC method [24, 38], where Student’s t-test and F-test at a 95% confidence level (*p* = 0.05) were calculated. The calculated t and F-values were lower than the tabulated values at the 95% confidence level, indicating no significant difference between the proposed method and the reference method. This confirms the accuracy and precision of the developed models (Table 2).

**Table 2:** Quantitative assay of Aztreonam and meropenem in their medications with statistical evaluation of the obtained results with a reported HPLC method

Parameter	PLS		GA-PLS		ANN		Reported method ^b^	
	AZM	MPM	AZM	MPM	AZM	MPM	AZM	MPM
Mean R% ^a^	95.99	97.87	99.46	99.87	97.46	99.42	98.34	98.02
SD ^**a**^	1.913	1.946	1.085	1.409	1.535	1.410	1.354	1.520
Variance ^**a**^	3.660	3.787	1.177	1.985	2.356	1.988	1.833	2.310
n	5						5	
Student t-test ^c^ (2.306)	2.242	0.136	1.443	1.996	0.961	1.510		
F-test ^c^ (6.39)	1.996	1.639	1.557	0.859	1.285	0.861		

^a^Average of five determinations

^b^A reversed-phase HPLC system equipped with a C_18_ column (150 × 4.6 mm, 5 μm) was used for the chromatographic separation of AZM and MPM. The mobile phase consisted of 20 mM phosphate buffer (pH 2.8) and acetonitrile in a ratio of 85:15 (v/v) under isocratic conditions. The flow rate and detection were set at 1.0 mL/min and 298 nm, respectively. The injection volume was 20 µL, and the total run time was 10 min

^c^The parentheses values signify the tabulated two-tailed values at *p* = 0.05

*AZM: aztreonam, MPM: meropenem

### Greenness evaluation via different assessment tools

#### SDS and spider radar

A multi-criteria SDS-based approach was employed to score solvents on toxicity, flammability, environmental impact, volatility/exposure risk, and corrosiveness. Each criterion was scored from 1 (least favorable) to 10 (most favorable) and normalized to obtain a Greenness Index. Visualization was achieved through a spider radar chart to illustrate individual solvent ability and composite diagrams (circular gauge and star plot) to depict overall sustainability. The chart enables an intuitive comparison and facilitates informed solvent selection.

**Fig. 7:**
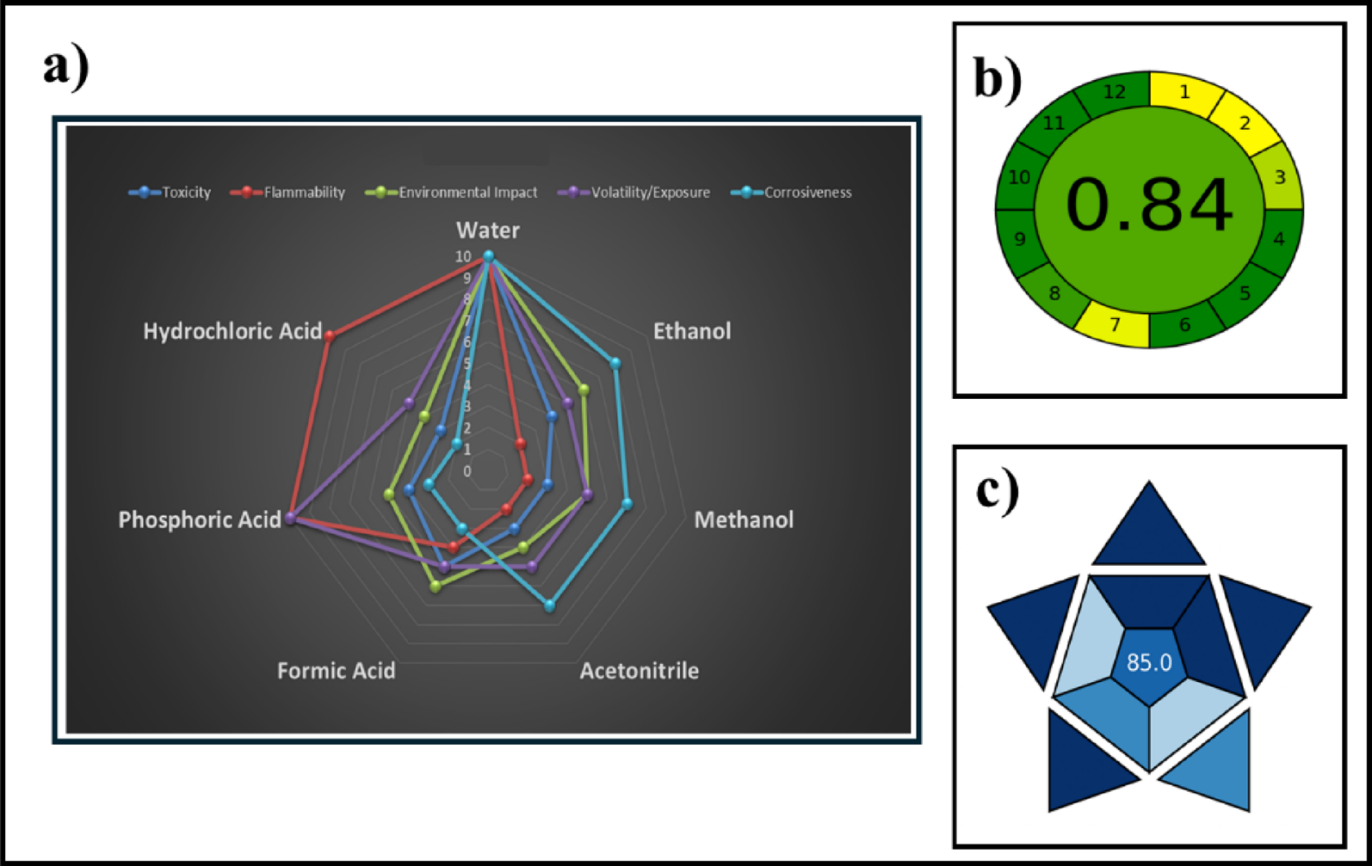
Greenness and sustainability assessment of the proposed analytical method using different evaluation tools: **a** Spider (radar) chart representing the SDS sustainability score for solvents based on multiple criteria; **b** AGREE diagram showing the overall compliance of the method with the 12 principles of Green Analytical Chemistry; and **c** BAGI diagram illustrating the method’s sustainability score, emphasizing its eco-friendly profile

Figure 7a provides a visual representation of solvent comparison using radar and composite diagrams, highlighting the necessity of adopting more sustainable alternatives, such as water, in analytical workflows. The figure illustrates the evaluations for the used solvent (water) compared to reported solvents for AZM and MPM determination, such as ethanol, methanol, acetonitrile, phosphoric acid, formic acid, and hydrochloric acid. Water exhibited the largest polygon compared to the constricted shapes of hazardous solvents, reflecting maximum scores across all safety and environmental categories, whereas acetonitrile and hydrochloric acid displayed restricted areas, indicating high toxicity, volatility, and corrosiveness. Ethanol and methanol showed intermediate activity, primarily limited by flammability concerns and moderate toxicity. This visualization emphasizes the superiority of water-based analytical methods for greenness compliance, highlighting its prominence as an ideal green solvent.

#### AGREE and BAGI assessment

The greenness of the proposed analytical method was assessed using the AGREE metric, a holistic tool based on the 12 principles of Green Analytical Chemistry (GAC). AGREE evaluates the method through a radial diagram that visually represents its environmental efficiency, providing a score between 0 (least green) and 1 (most green). The proposed method achieved an AGREE score of 0.84 (Fig. 7b), reflecting its compliance with several green chemistry principles such as minimal reagent consumption, reduced energy requirements, and generation of negligible waste. The results confirm that the method offers a sustainable and environmentally friendly alternative compared to conventional approaches. The Green Analytical Procedure Index (BAGI) was also applied to evaluate the method’s sustainability. BAGI assesses the greenness of analytical procedures by scoring various factors such as sample preparation, energy consumption, waste generation, and occupational hazards. The developed method obtained a BAGI score of 85.0 (Fig. 7c), reflecting its low environmental impact and high safety profile. The favorable BAGI results support the claim that the proposed procedure aligns with the principles of Green Analytical Chemistry and promotes a sustainable analytical workflow.

To further support the greenness of the developed method, its functionality was compared with previously reported analytical procedures for the determination of AZM and MPM. The proposed method demonstrates excellent compliance with green and sustainable chemistry principles, as confirmed by high AGREE (0.84), BAGI (85.0), and favorable SDS scores (Supp. Table S6). These scores not only confirm the eco-friendly profile of the proposed chemometric methodology but also demonstrate its industrial and regulatory relevance, reflecting strong compliance regarding solvent minimization, energy efficiency, and waste reduction. These attributes align with the international recommendations encouraging sustainable analytical procedures in pharmaceutical development and routine quality control.

### Summary of findings and methodological novelty

By systematically comparing MC, LHS, and SS with PLS, GA-PLS, ANN, and a new GA-ICOMP-PLS algorithm, the work establishes clear evidence that sampling design plays a critical role in model accuracy and robustness. The SS-ANN combination demonstrated superior predictive performance, while LHS-GA-PLS further enhanced model reliability. Incorporating the GA-ICOMP-PLS algorithm significantly strengthened model predictability, achieving an improvement of 35.1% – 63.6% in performance over standard PLS, underscoring the effectiveness of combining GA-based variable selection with ICOMP criteria to eliminate redundant wavelengths and enhance calibration robustness.

To rigorously validate model reliability, nested cross-validation and external validation confirmed unbiased predictive capability, Y-randomization verified the absence of chance correlation, and AD analysis ensured all samples were within the reliable prediction space. Additionally, noise-profile and sampling-sensitivity evaluations demonstrated stable signal capture and resilience to spectral variability, reinforcing model robustness under practical analytical conditions. Collectively, these results demonstrate that the optimized sampling-modeling strategy yields highly accurate predictions with reduced error metrics and reliable calibration behavior. In addition to methodological innovation, the study delivers a green, efficient, and resource-conscious analytical alternative, supported by strong AGREE and BAGI scores.

A literature review confirms that no prior work has applied chemometric or multivariate calibration tools for the simultaneous analysis of AZM and MPM, where traditional methods predominantly rely on HPLC and conventional UV-Vis techniques. Thus, the proposed strategy introduces a novel and environmentally conscious analytical paradigm for β-lactam quantification, offering superior precision, efficiency, and practical applicability compared to reported instrumental approaches.

## Conclusion

The current study highlights the important influence of different sampling techniques on the accuracy and predictive ability of various chemometric models in multivariate pharmaceutical analysis of AZM and MPM. Through the integration of MC, LHS, and SS methods with PLS, GA-PLS, and ANN models, it was found that the structure and uniformity of the calibration and validation datasets play a significant role in enhancing models’ robustness and predictive capabilities. Among the constructed models, it was found that the ANN model combined with the SS technique achieved the highest accuracy and generalizability compared to the classical PLS model. Moreover, the GA-PLS model combined with the LHS technique was also able to provide a strong ability for linear calibration scenarios, while MC combined with PLS exhibited lower efficiency due to data clustering effects and uneven space coverage. Nested cross-validation and external validation frameworks were carried out, confirming that the models are robust and free from information leakage. While Y-randomization tests confirmed that the predictive performance is genuine and not a result of chance correlation. The AD assessment showed that all samples lie within the reliable prediction space, with no influential outliers detected. Moreover, the hybrid GA-ICOMP-PLS method was developed to enhance the predictive powers and generalization of the constructed models. These results emphasize the significance of using appropriate sampling techniques during model development for building more reliable and efficient chemometric tools in pharmaceutical quality control and spectral data analysis. Additionally, the greenness and sustainability of the proposed analytical method were comprehensively evaluated using AGREE, BAGI, and SDS assessment tools. The method achieved high scores across all evaluations, with an AGREE score of 0.84, a BAGI score of 85.0, and favorable SDS values for the solvents employed. These findings confirm that the developed procedure not only meets analytical requirements but also aligns with the principles of Green Analytical Chemistry by reducing reagent consumption, minimizing environmental hazards, and promoting safer laboratory practices. Consequently, this method represents an environmentally responsible and sustainable alternative to conventional approaches.

## Supplementary Information

Supplementary Material 1

## Data Availability

All relevant data used in this study are available from the corresponding author upon reasonable request.
