# Supplementary material for: Hybrid sampling chemometric strategy for accurate and sustainable quantification of co-administered β-lactam antibiotics
Source: BMC Chem. 2025 Dec 24;20(1):18. doi: 10.1186/s13065-025-01695-9 (PMC12837029; doi:10.1186/s13065-025-01695-9)
Supplement: Supplementary file 1 — Supplementary Material 1 [file 13065_2025_1695_MOESM1_ESM.docx]

**Supplementary information for**

**“Hybrid Sampling Chemometric Strategy for Accurate and Sustainable Quantification of Co-Administered β-Lactam Antibiotics"**

Shymaa S. Soliman^a,1*^, Mona A. abdelrahman^a,2^

^a^ Analytical Chemistry Department, Faculty of Pharmacy, October 6 University, October 6 City, Giza, 12858, Egypt

** Corresponding author email:

^1^ [shimaasayed@o6u.edu.eg](mailto:shimaasayed@o6u.edu.eg)

^2^ mona.alkhateb89@gmail.com

* Mailing address: October 6 University, Faculty of Pharmacy, Giza, 12858, Egypt.

* Tel:

^1^ +20-1004581866

^2^ +20-1090143451

^1^ ORCID ID: 0000-0002-0554-0423

^2^ ORCID ID: 0009-0006-5264-7289

# **Sampling techniques**

## Monte Carlo technique (MC)

Monte Carlo is a sampling process that is used for numerical integration and variance estimation. It is based on generating a large number of random representative datasets by using statistical inference to evaluate desired quantities. It assumes a uniform distribution and samples independently across the experimental range. Four main steps are involved during MC integration (1). The first step is defining the range of the analyzed domain, which could be a physical space, a set of parameters, or a range of values for a variable. Mathematically, each variable (𝑥_𝑖_) is selected randomly from a uniform distribution; **𝑥_𝑖 ∼_ υ (a_𝑖_, b_𝑖_)**, where **a_𝑖_** and **b_𝑖_** are the lower and upper limits of the variable range. Then, random points within the defined domain were generated according to the distribution probability, which can be uniform, normal, or any other distribution that reflects different inputs. Afterward, deterministic calculations were performed for each random sample, followed by aggregating the results to achieve deep insights into the overall behavior of the model or the encountered problem.

## Latin Hypercube Sampling (LHS)

Latin hypercube sampling is an arithmetical technique that was introduced in the late 1970s and was used to generate stratified samples from multidimensional distributions (2). It divides each variable's range (*d*) into intervals of equal segments (*ƞ*) to ensure comprehensive coverage of the entire parameter space. Each segment is then sampled once and represented as: $i= \left[ \frac{i-1}{n}, \frac{i}{n} \right]$ for *i* = 1, 2, …..., *ƞ*. Then the sample values are randomly shuffled across dimensions (*d*) to guarantee a unique combination of each segment. Using this structured sampling strategy reduces sample clustering and improves the coverage of the input space.

## Sobol Sequence (SS)

Sobol sequence is a quasi-random low-discrepancy sequence that was first introduced by the Russian mathematician Ilya Sobol (3). They are designed to fill the design space more uniformly [0,1]*^d^* than random sampling using specific mathematical algorithms, minimizing "clumps" or "empty spaces" in the sampled points. Unlike other random techniques, SS uses a deterministic algorithm where each point index (*i*) in the SS is generated using binary index representations, where each binary digit (𝑏_𝑘_) is used in a bitwise operations (XOR) on precomputed direction numbers (υ*_k, j_*) which are specific to each dimension (𝑗) (4,5). Then, a quasi-random sequence originates without repetition as $x_{i}^{(j)}= b_{1}$υ*_1, j_* ^⊕^_,_ $b_{2}$υ*_2_, _j_* ^⊕^*_,_* _……,_ where ⊕ is the bitwise XOR. These direction numbers are obtained from primitive polynomials over a finite range, confirming uniformity across multiple dimensions.

**Supplementary material (Table S1).** Concentrations of aztreonam and meropenem in the calibration and validation sets for the multivariate calibration models.

| **Mixture No.** | **Concentrations (µg mL^-1^)** | | | | | | | |
| --- | --- | --- | --- | --- | --- | --- | --- | --- |
|  | **Calibration Set** | | **Validation Set** | | | | | |
|  |  |  | **Monte Carlo** | | **Latin Hypercube** | | **Sobol Sequence** | |
|  | **AZM** | **MPM** | **AZM** | **MPM** | **AZM** | **MPM** | **AZM** | **MPM** |
| 1 | 20.00 | 20.00 | 24.86 | 10.69 | 19.63 | 16.10 | 10.00 | 10.00 |
| 2 | 20.00 | 10.00 | 17.84 | 18.77 | 21.70 | 23.17 | 20.00 | 20.00 |
| 3 | 10.00 | 10.00 | 23.11 | 17.63 | 12.07 | 12.49 | 15.00 | 25.00 |
| 4 | 10.00 | 30.00 | 13.42 | 25.31 | 23.58 | 13.44 | 25.00 | 15.00 |
| 5 | 30.00 | 15.00 | 24.12 | 25.90 | 18.14 | 25.70 | 12.50 | 22.50 |
| 6 | 15.00 | 30.00 | 10.64 | 13.74 | 11.49 | 22.02 | 22.50 | 12.50 |
| 7 | 30.00 | 20.00 | 15.54 | 19.80 | 15.73 | 29.25 | 17.50 | 17.50 |
| 8 | 20.00 | 15.00 | 10.92 | 18.91 | 17.62 | 17.01 | 27.50 | 27.50 |
| 9 | 15.00 | 15.00 | 11.94 | 22.93 | 26.77 | 10.54 | 11.25 | 28.75 |
| 10 | 15.00 | 25.00 | 26.47 | 24.19 | 27.19 | 24.29 | 21.25 | 18.75 |
| 11 | 25.00 | 30.00 | 23.90 | 25.09 | 13.55 | 27.30 | 16.25 | 13.75 |
| 12 | 30.00 | 25.00 | 16.34 | 15.52 | 29.51 | 18.81 | 26.25 | 23.75 |
| 13 | 25.00 | 20.00 | 29.00 | 23.59 | 23.92 | 19.72 | 13.75 | 16.25 |
| 14 | 20.00 | 30.00 |  |  |  |  |  |  |
| 15 | 30.00 | 30.00 |  |  |  |  |  |  |
| 16 | 30.00 | 10.00 |  |  |  |  |  |  |
| 17 | 10.00 | 25.00 |  |  |  |  |  |  |
| 18 | 25.00 | 10.00 |  |  |  |  |  |  |
| 19 | 10.00 | 20.00 |  |  |  |  |  |  |
| 20 | 20.00 | 25.00 |  |  |  |  |  |  |
| 21 | 25.00 | 25.00 |  |  |  |  |  |  |
| 22 | 25.00 | 15.00 |  |  |  |  |  |  |
| 23 | 15.00 | 10.00 |  |  |  |  |  |  |
| 24 | 10.00 | 15.00 |  |  |  |  |  |  |
| 25 | 15.00 | 20.00 |  |  |  |  |  |  |

^*^ AZM: aztreonam, MPM: meropenem.

**Supplementary material (Table S2).** Configuration of the Genetic Algorithm parameters.

| **Parameter** | **Value** |
| --- | --- |
| Population size | 30 |
| Maximum generations | 50 |
| Mutation rate | 0.005 |
| The number of variables in a window (window width) | 5 |
| Percent of population (% of convergence) | 100 |
| % Wavelengths used at initiation | 50 |
| Crossover type | Double |
| Maximum number of latent variables | 3 |
| Cross-validation | Random |
| Number of subsets to divide data into for cross-validation | 4 |
| Number of iterations for cross-validation at each generation | 3 |

**Supplementary material (Table S3).** Validation set analysis using established chemometric models.

|  | **Concentrations (μg mL^-1^)** | | **PLS** | | **GA-PLS** | | **ANN** | |
| --- | --- | --- | --- | --- | --- | --- | --- | --- |
|  |  |  | **Recovery %** | | **Recovery %** | | **Recovery %** | |
|  | **AZM** | **MPM** | **AZM** | **MPM** | **AZM** | **MPM** | **AZM** | **MPM** |
| **Monte Carlo** | 24.86 | 10.69 | 100.29 | 98.33 | 101.37 | 99.50 | 101.77 | 98.56 |
|  | 17.84 | 18.77 | 98.31 | 98.26 | 97.80 | 98.39 | 99.48 | 97.33 |
|  | 23.11 | 17.63 | 98.70 | 101.29 | 99.04 | 99.15 | 98.61 | 100.28 |
|  | 13.42 | 25.31 | 96.29 | 95.76 | 101.63 | 98.00 | 100.88 | 99.58 |
|  | 24.12 | 25.90 | 97.69 | 96.79 | 97.36 | 97.91 | 97.92 | 99.46 |
|  | 10.64 | 13.74 | 96.40 | 97.72 | 98.91 | 101.90 | 97.97 | 98.98 |
|  | 15.54 | 19.80 | 96.43 | 100.23 | 96.11 | 96.34 | 98.68 | 97.35 |
|  | 10.92 | 18.91 | 98.73 | 96.07 | 98.08 | 99.36 | 99.00 | 98.30 |
|  | 11.94 | 22.93 | 99.54 | 97.20 | 100.74 | 99.70 | 102.42 | 98.39 |
|  | 26.47 | 24.19 | 96.47 | 101.95 | 98.11 | 102.20 | 99.24 | 101.79 |
|  | 23.90 | 25.09 | 96.81 | 95.86 | 98.27 | 101.97 | 98.69 | 99.58 |
|  | 16.34 | 15.52 | 101.86 | 98.04 | 98.97 | 102.57 | 100.81 | 100.63 |
|  | 29.00 | 23.59 | 98.78 | 100.59 | 101.98 | 101.61 | 101.29 | 102.46 |
|  | **Mean** | | 98.18 | 98.31 | 99.11 | 99.89 | 99.75 | 99.44 |
|  | **RSD%** | | 1.756 | 2.127 | 1.818 | 1.985 | 1.503 | 1.563 |
|  | **RMSEP ^a^** | | 0.4719 | 0.5890 | 0.3904 | 0.4028 | 0.2844 | 0.3266 |
| **Latin Hypercube** | 19.63 | 16.10 | 101.23 | 100.80 | 101.58 | 100.59 | 100.65 | 99.98 |
|  | 21.70 | 23.17 | 98.60 | 99.97 | 97.47 | 98.69 | 97.65 | 101.59 |
|  | 12.07 | 12.49 | 98.96 | 98.81 | 97.14 | 97.90 | 98.52 | 98.08 |
|  | 23.58 | 13.44 | 99.46 | 98.39 | 99.79 | 98.89 | 99.69 | 100.76 |
|  | 18.14 | 25.70 | 99.47 | 99.57 | 96.16 | 98.86 | 98.05 | 98.25 |
|  | 11.49 | 22.02 | 97.77 | 100.46 | 100.78 | 99.88 | 100.00 | 101.04 |
|  | 15.73 | 29.25 | 97.40 | 97.94 | 96.35 | 98.61 | 98.49 | 100.01 |
|  | 17.62 | 17.01 | 98.67 | 99.54 | 101.10 | 101.34 | 99.26 | 101.43 |
|  | 26.77 | 10.54 | 97.91 | 98.20 | 98.39 | 101.68 | 96.66 | 99.96 |
|  | 27.19 | 24.29 | 99.70 | 102.55 | 99.40 | 102.21 | 101.09 | 99.82 |
|  | 13.55 | 27.30 | 97.61 | 98.56 | 99.71 | 99.61 | 98.25 | 97.21 |
|  | 29.51 | 18.81 | 99.12 | 98.79 | 100.43 | 96.60 | 99.99 | 98.58 |
|  | 23.92 | 19.72 | 100.53 | 98.45 | 100.95 | 98.32 | 98.27 | 99.07 |
|  | **Mean** | | 98.96 | 99.39 | 99.17 | 99.47 | 98.97 | 99.68 |
|  | **RSD%** | | 1.155 | 1.309 | 1.890 | 1.626 | 1.284 | 1.368 |
|  | **RMSEP ^a^** | | 0.3572 | 0.3218 | 0.2758 | 0.3034 | 0.3535 | 0.3042 |
| **Sobol Sequences** | 10.00 | 10.00 | 97.12 | 98.42 | 98.83 | 100.61 | 100.00 | 100.00 |
|  | 20.00 | 20.00 | 99.94 | 100.42 | 99.57 | 102.91 | 102.50 | 98.75 |
|  | 15.00 | 25.00 | 100.08 | 100.91 | 98.05 | 100.23 | 100.00 | 99.00 |
|  | 25.00 | 15.00 | 98.68 | 99.17 | 95.92 | 101.81 | 102.00 | 100.33 |
|  | 12.50 | 22.50 | 97.50 | 101.59 | 101.34 | 101.22 | 100.00 | 99.55 |
|  | 22.50 | 12.50 | 99.35 | 95.48 | 97.42 | 99.24 | 99.59 | 96.09 |
|  | 17.50 | 17.50 | 101.11 | 96.64 | 99.25 | 102.97 | 99.94 | 99.08 |
|  | 27.50 | 27.50 | 98.44 | 98.68 | 99.51 | 99.84 | 100.00 | 100.91 |
|  | 11.25 | 28.75 | 99.67 | 97.54 | 102.09 | 97.97 | 102.22 | 100.00 |
|  | 21.25 | 18.75 | 99.17 | 97.23 | 97.51 | 99.26 | 98.82 | 100.00 |
|  | 16.25 | 13.75 | 97.44 | 98.57 | 97.84 | 97.17 | 98.46 | 101.82 |
|  | 26.25 | 23.75 | 100.02 | 99.41 | 101.50 | 101.51 | 101.71 | 100.00 |
|  | 13.75 | 16.25 | 96.21 | 97.47 | 96.58 | 102.89 | 101.82 | 97.23 |
|  | **Mean** | | 98.82 | 98.58 | 98.88 | 100.59 | 100.54 | 99.44 |
|  | **RSD%** | | 1.437 | 1.770 | 1.937 | 1.858 | 1.332 | 1.501 |
|  | **RMSEP ^a^** | | 0.2791 | 0.3945 | 0.4277 | 0.3547 | 0.2719 | 0.2369 |

^a^ Root mean square error of prediction.

^*^ AZM: aztreonam, MPM: meropenem.

**Supplementary material (Table S4).** Predictive performance of chemometric models for aztreonam and meropenem.

| **Model** | **Drug** | **RMSECV (95% CI)** | \|  \| \| --- \|   **MSEP (95% CI)** | **Latent Variables / Neurons** | **Selected variables** |
| --- | --- | --- | --- | --- | --- | --- |
| **PLS** | **AZM** | \|  \| \| --- \|   1.025 [0.670–1.400] | 1.650 [0.769–2.314] | 6 | All |
|  | **MPM** | \|  \| \| --- \|   0.548 [0.289–0.813] | 0.755 [0.431–1.005] | 3 | All |
| **GA-PLS** | **AZM** | \|  \| \| --- \|   0.584 [0.365–0.804] | 1.650 [0.769–2.314] | 6 | 50 |
|  | **MPM** | \|  \| \| --- \|  \|  \| \| --- \|   0.503 [0.289–0.813] | 0.633 [0.431–1.005] | 3 | 42 |
| **ANN** | **AZM** | \|  \| \| --- \|   1.907 [1.378–2.436] | 1.912 [1.350–2.470] | 6 | All |
|  | **MPM** | 1.3099 [0.8909, 1.7288] | 1.4418 [0.5211 – 1.3048] | 6 | All |

**Supplementary material (Table S5).** Downsampling and spectral region assessments for comparable RMSEP/R² values for the PLS-MC model at different binning steps and scanning regions.

| **Drugs** | **Wavelength step (nm)/ Spectral range (nm)** | **RMSEP** | **R^2^** |
| --- | --- | --- | --- |
| **AZM** | 0.1 | 0.4719 | 0.9970 |
|  | 0.5 | 0.4593 | 0.9841 |
|  | 1.0 | 0.4211 | 0.9964 |
| **MPM** | 0.1 | 0.5890 | 0.9902 |
|  | 0.5 | 0.5771 | 0.9842 |
|  | 1.0 | 0.5123 | 0.9880 |
| **AZM** | 200.0 – 335.0 | 0.4719 | 0.9970 |
|  | 190.0 ̶ 340.0 | 0.3999 | 0.9881 |
|  | 205.0 ̶ 400.0 | 0.4220 | 0.9741 |
| **MPM** | 200.0 – 335.0 | 0.5890 | 0.9902 |
|  | 190.0 ̶ 340.0 | 0.4886 | 0.9932 |
|  | 205.0 ̶ 400.0 | 0.5120 | 0.9741 |

**Supplementary material (Table S6).** Comparison of the greenness profiles of the proposed and reported chemometric models using the assessment tools.

| **Reference** | **Greenness evaluation** | **Reference** | **Greenness evaluation** |
| --- | --- | --- | --- |
| **Proposed method** | 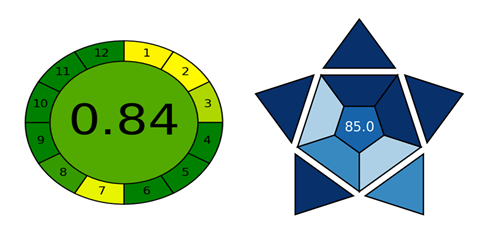 | | |
| (6) | **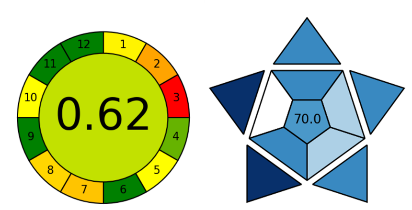** | (7) | **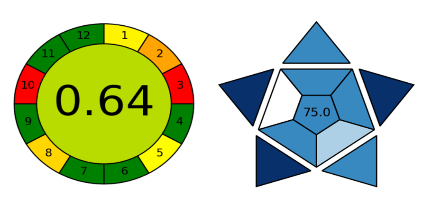** |
| (8) | **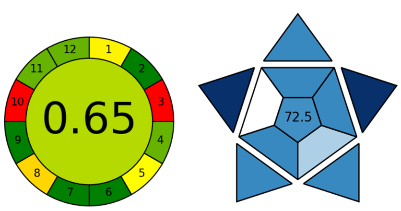** | (9) | **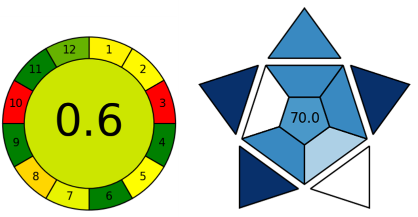** |
| (10) | **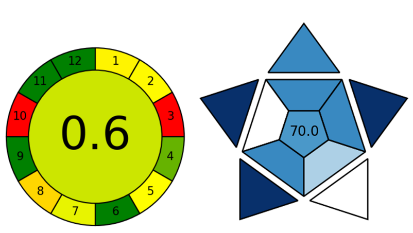** | (11) | **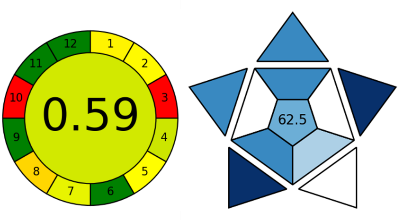** |
| (12) | **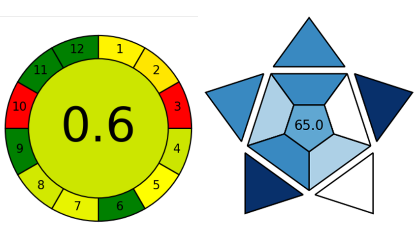** | (13) | **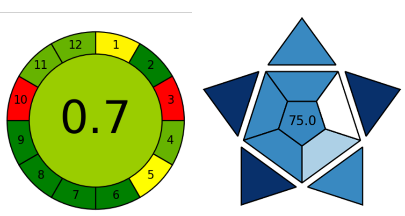** |
| (14) | **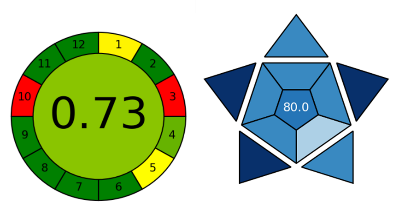** | (15) | **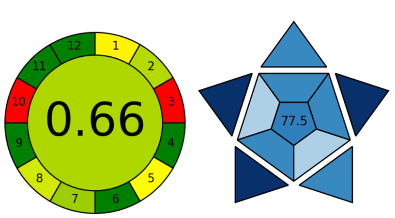** |
| (16) | **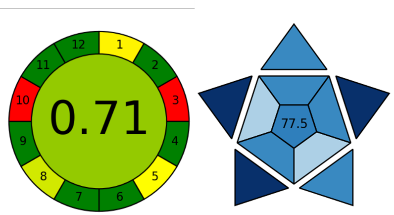** | (17) | **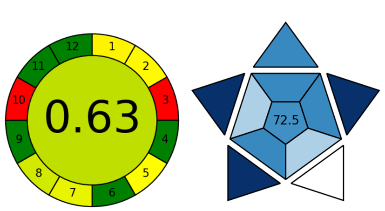** |
| (18) | **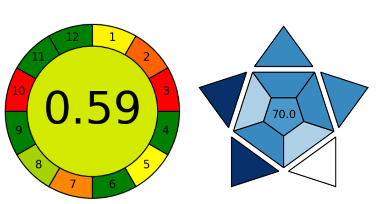** |  |  |


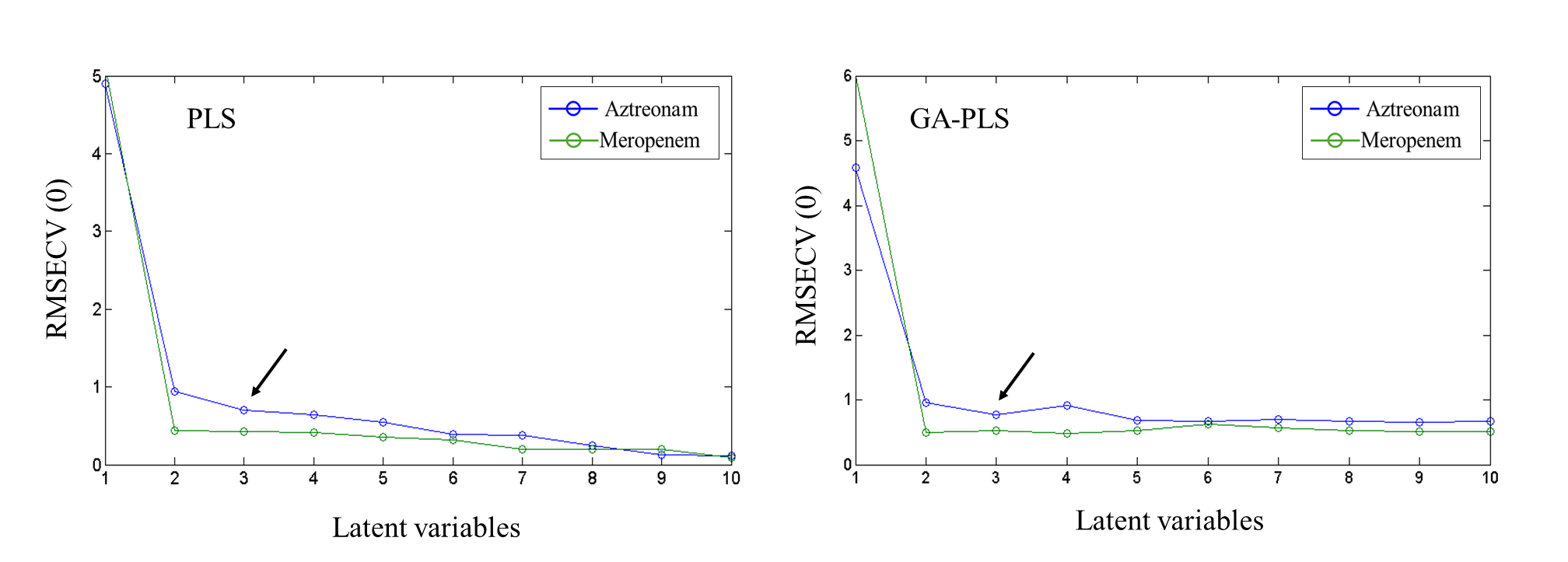


**Supplementary material (Figure S1).** RMSECV plot of the cross-validation results of the calibration set to determine the optimum latent variables used to construct the developed models.


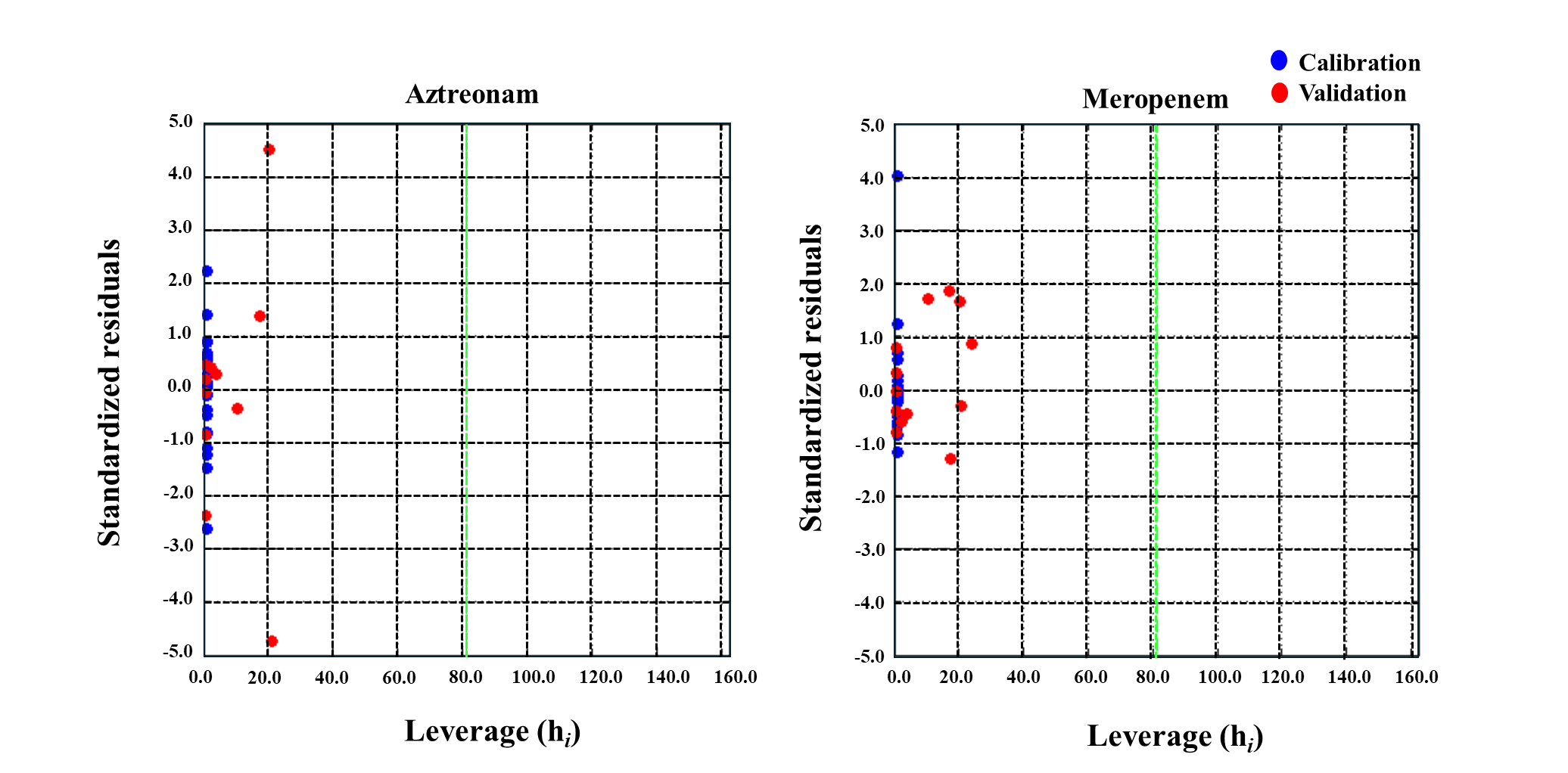


**Supplementary material (Figure S2).** Williams plots illustrating the applicability domain (AD) of the developed PLS-based models for aztreonam and meropenem. The critical leverage value (h^*^) is represented by the green dashed line, defining the model’s reliable prediction boundary. All samples fall within the defined domain, confirming model robustness and the absence of influential outliers.


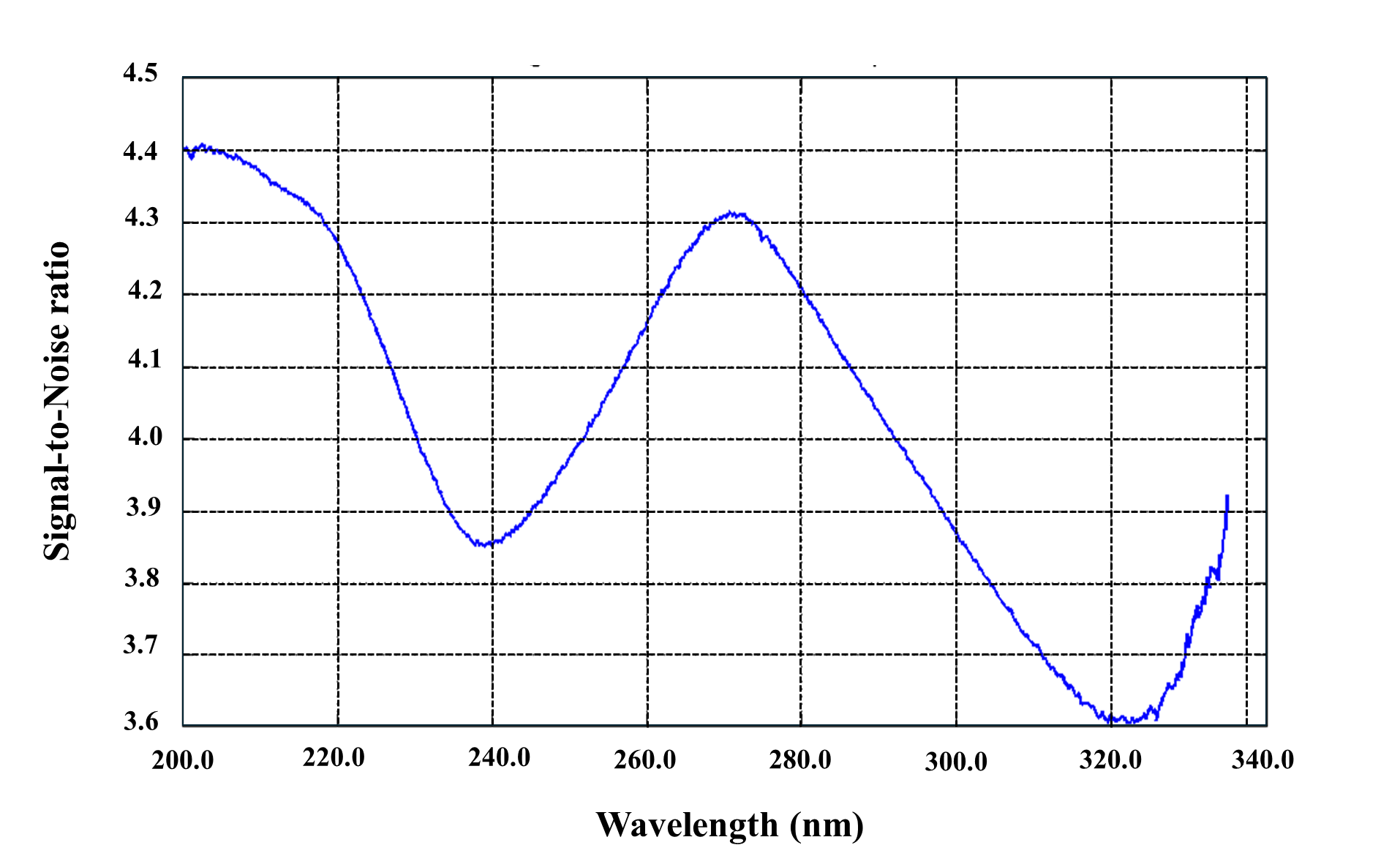


**Supplementary material (Figure S3).** Signal-to-noise ratio distribution across the UV spectra (200.0 – 335.0 nm), showing stable and high signal-to-noise levels without abrupt noise spikes, confirming data quality and suitability of the wavelength range for chemometric modeling.

**References**

1. Song C, Kawai R. Monte Carlo and variance reduction methods for structural reliability analysis: A comprehensive review. Probabilistic Eng Mech. 2023;73:103479. Available from: https://08101902b-1103-y-https-www-sciencedirect-com.mplbci.ekb.eg/science/article/pii/S0266892023000681?via%3Dihub#sec3

2. McKay MD, Beckman RJ, Conover WJ. Comparison of Three Methods for Selecting Values of Input Variables in the Analysis of Output from a Computer Code. Technometrics. 1979;21(2):239–45. Available from: http://www.tandfonline.com/doi/abs/10.1080/00401706.1979.10489755

3. Sobol’ I. On the distribution of points in a cube and the approximate evaluation of integrals. USSR Comput Math Math Phys. 1967;7(4):86–112. Available from: https://www.sciencedirect.com/science/article/abs/pii/0041555367901449

4. Joe S, Kuo FY. Remark on algorithm 659. ACM Trans Math Softw. 2003;29(1):49–57. Available from: https://dl.acm.org/doi/10.1145/641876.641879

5. Joe S, Kuo FY. Constructing Sobol Sequences with Better Two-Dimensional Projections. SIAM J Sci Comput. 2008;30(5):2635–54. Available from: /doi/pdf/10.1137/070709359?download=true

6. Figueiredo AL, Kogawa AC, Salgado HRN. Development and validation of an ecological, new and rapid stability-indicating high performance liquid chromatography for quantitative determination of aztreonam in lyophilized powder for injection. Drug Anal Res. 2017 Aug 28;1(1):24–30. Available from: https://seer.ufrgs.br/index.php/dar/article/view/73755

7. Bhattacharyya S, Sogali PS and BS. Validation of RP-HPLC Method and UV Spectrophotometric Method for the Quantitative Determination of Aztreonam in Bulk and Pharmaceutical Dosage Form. RGUHS J Pharm Sci. 2018;8(4):120–6. Available from: https://journalgrid.com/view/article/rjps/1134

8. Prakash V, Suresh J, Konari SN. RP-HPLC Forced Degradation Studies of Aztreonam in Pharmaceutical Dosage Form. Pharm Methods. 2017;9(1):40–4. Available from: https://www.google.com/search?q=RP-HPLC+Forced+Degradation+Studies+of+Aztreonam+in%0D%0APharmaceutical+Dosage+Form+&sca_esv=088c43baef1148eb&biw=1536&bih=695&sxsrf=AE3TifMA_exqsRsbvlZ1i4hq3XV_bUne0A%3A1750584376954&ei=OMxXaI-BOpCki-gP46_e8QI&ved=0ahUKEwiP

9. Leme de Figueiredo A, Regina Nunes Salgado H. Development and Validation of a Ultraviolet (UV) Spectrophotometric Method for Determination of aztreonam in Pharmaceutical Products. EC Microbiol. 2017;8(6):305–16. Available from: https://ecronicon.net/assets/ecmi/pdf/ECMI-08-00264.pdf

10. Qassim AW. Spectrophotometric Method for the Estimation of Meropenem in Pure and in Market Formulation Meronem. ResearchgateNet. 2015;7(4):59–67.

11. Al-Abbasi MA, Samarrai ET Al, Alwan LH. Spectrophotometric determination of meropenem trihydrate in pharmaceutical preparations by oxidative coupling reaction with mefenamic acid using sodium nitroprusside. Samarra J Pure Appl Sci. 2024;6(2/2):85–99. Available from: https://www.sjpas.com/index.php/sjpas/article/view/663

12. Fayed AS, Youssif RM, Salama NN, Elzanfaly ES, Hendawy HAM. Utility of Silver-nanoparticles for Nano Spectrofluorimetric Determination of Meropenem and Ertapenem: Bio-analytical Validation. Spectrochim Acta Part A Mol Biomol Spectrosc [Internet]. 2021;262:120077. Available from: https://linkinghub.elsevier.com/retrieve/pii/S1386142521006545

13. Verdier M-C, Tribut O, Tattevin P, Le Tulzo Y, Michelet C, Bentué-Ferrer D. Simultaneous Determination of 12 β-Lactam Antibiotics in Human Plasma by High-Performance Liquid Chromatography with UV Detection: Application to Therapeutic Drug Monitoring. Antimicrob Agents Chemother. 2011;55(10):4873–9. Available from: /doi/pdf/10.1128/aac.00533-11?download=true

14. Van Vooren S, Verstraete AG. A sensitive and high‐throughput quantitative liquid chromatography high‐resolution mass spectrometry method for therapeutic drug monitoring of 10 β ‐lactam antibiotics, linezolid and two β ‐lactamase inhibitors in human plasma. Biomed Chromatogr. 2021 Jul 2;35(7):e5092. Available from: /doi/pdf/10.1002/bmc.5092

15. Fayed AS, Youssif RM, Salama NN, Hendawy HA, Elzanfaly ES. Two-wavelength manipulation stability-indicating spectrophotometric methods for determination of meropenem and ertapenem: greenness consolidation and pharmaceutical product application. Chem Pap. 2019];73(11):2723–36. Available from: https://link.springer.com/article/10.1007/s11696-019-00824-8

16. El-Kosasy AM, Abdel-Aziz O, Youssif RM, Salama NN. Feasible oxidative degradates separation with instant quantification of two non-classical β-lactams by derivative UV spectrophotometry and TLC densitometry. Chem Pap. 2020;74(10):3557–67. Available from: https://link.springer.com/10.1007/s11696-020-01181-7

17. Elzanfaly ES, Youssif RM, Salama NN, Fayed AS, Hendawy HAM, Salem MY. Zero and second-derivative synchronous fluorescence spectroscopy for the quantification of two non-classical β-lactams on pharmaceutical vials: application to stability studies. Luminescence. 2017;32(8):1517–27.

18. Hendawy HAM, Youssif RM, Salama NN, Fayed AS, Salem MY. Challenge Approach of an Inexpensive Electrochemical Sensor for Rapid Selective Determination of two Non‐classical β‐Lactams in Presence of Different Degradants and Interference Substances. Electroanalysis. 2017 D;29(12):2708–18. Available from: https://analyticalsciencejournals.onlinelibrary.wiley.com/doi/10.1002/elan.201700431
